# Disentangling the effects of resource level and temperature dependence on the performance of fish in different guilds

**DOI:** 10.1093/conphys/coag005

**Published:** 2026-01-28

**Authors:** Bass Dye, Myron A Peck, Karen E van de Wolfshaar, Anieke van Leeuwen

**Affiliations:** Department of Coastal Systems, NIOZ Royal Netherlands Institute for Sea Research, P.O. Box 59, 1790 AB, Den Burg, Texel, The Netherlands; Wageningen Marine Research, Wageningen University and Research, Herring Quay 1, 1976 CP, IJmuiden, The Netherlands; Groningen Institute for Evolutionary Life Sciences—GELIFES, University of Groningen, Nijenborgh 7, Groningen 9747 AG, The Netherlands; Department of Coastal Systems, NIOZ Royal Netherlands Institute for Sea Research, P.O. Box 59, 1790 AB, Den Burg, Texel, The Netherlands; Marine Animal Ecology Group, Department of Animal Sciences, Wageningen University & Research, Droevendaalsesteeg 1, Building 107, 6708 PB, Wageningen, The Netherlands; Wageningen Marine Research, Wageningen University and Research, Herring Quay 1, 1976 CP, IJmuiden, The Netherlands; Department of Coastal Systems, NIOZ Royal Netherlands Institute for Sea Research, P.O. Box 59, 1790 AB, Den Burg, Texel, The Netherlands

**Keywords:** Climate change, fecundity, growth, marine fish, metabolism, thermal performance

## Abstract

The ability to predict how fishes respond to changes in temperature and resource variability is paramount to developing sustainable management plans and for projecting the direct and indirect effects of climate change. We developed a versatile, physiological model capable of providing size-specific estimates of fish growth and fecundity across varying temperatures and resource levels. The model includes a mechanistic representation of individual-level life history processes across diverse biogeographic and functional fish guilds, using direct, species-specific parameter estimates. We demonstrate its application to five marine species (Atlantic cod, Atlantic herring, five-bearded rockling, European sprat and thinlip mullet), which differ in life history strategies and biogeographic distributions, but all rely on intertidal nursery habitats—areas particularly susceptible to anthropogenic change. In all simulations, resource availability had a stronger influence on fish performance than temperature. Nevertheless, the model also revealed how and why higher temperatures often decreased fitness and/or survival of specific types of species. We made no changes to the model structure for different species, and the resulting model predictions were not fitted but were based on eco-physiological first principles. Comparison between modelled and empirical data collected in the shallow Wadden Sea (southern North Sea) confirmed benefits of warming to thermophilic, range-expanding species, while core (established) species at their lower latitudinal limits of their distribution face local extirpation. The model allows insight into more variables than often reported from survey and monitoring efforts, such as reproductive output. The model’s broad applicability across a range of species, geographic regions and research objectives makes it valuable for generating knowledge needed to buttress actions aimed at addressing ecological and conservation challenges in a future climate.

## Introduction

Climate-driven warming and ocean acidification due to rising atmospheric carbon dioxide levels pose some of the most imminent threats to marine organisms (Hoegh-Guldberg and Bruno, 2010; Doney *et al.*, 2012; Poloczanska *et al.*, 2016; IPCC, 2023). Marine ectothermic organisms, such as fish, experience the globally warming seawater temperature directly. The warmer environment causes the metabolic rate of fish to increase, which alters their performance in terms of growth rates and reproductive output (Fry, 1947). In addition to these direct, physiological effects, fish species face changing trophic interactions and declining habitat suitability (Deutsch *et al.*, 2015; Pinsky *et al.*, 2019). Slow-growing fish species that reach large maximum sizes face considerable global pressures (Daufresne *et al.*, 2009; Fernandes *et al.*, 2017). Equally concerning, however, are the high anthropogenic pressures experienced in coastal zones—water bodies that are indispensable as nurseries for early life stages of both fast- and slow-growing fish species (Brown *et al.*, 2018; McLean *et al.*, 2019; Matić-Skoko *et al.*, 2020).

Coastal marine ecosystems have among the largest human footprints of all marine ecosystems (Reimann *et al.*, 2023) and are under serious threat from ongoing environmental and anthropogenic changes (Harley *et al.*, 2006; Lotze *et al.*, 2006). These are often habitats used by early fish life stages, due to high resource productivity and lowered predation risk (Elliott *et al.*, 2007). In these shallow systems, the increase in seawater temperature can be higher compared to deeper shelf seas and oceans (Harley *et al.*, 2006), and these coastal habitats lack depth refuges that species could use to avoid extreme temperatures (Dulvy *et al.*, 2008). Together with the direct physiological responses of fish to increased seawater temperatures, indirect effects of warming include changes in the resource environment experienced by fish (Wiltshire *et al.*, 2010; Defriez *et al.*, 2016). The combination of direct and indirect responses to extreme temperatures lies at the core of changes in population dynamics and plays a crucial role in the structure (e.g. species composition) and function of coastal communities (Dahlke *et al.*, 2020; Peck *et al.*, 2021). However, the temperature-dependent changes in resource availability and the subsequent food-dependent responses in fish are harder to predict than direct thermal responses in physiology (Ouellet *et al.*, 2025). The well-known direct thermal responses in physiology have been shown to underlie general trends of declining body sizes in response to climate warming (reviewed in Ohlberger, 2013), but the extent to which concurrent changes in food availability influence these responses is unclear.

To build models providing predictions of the physiological performance of fish, there are two broadly recognized approaches. On the one hand, the bioenergetics approach assumes an energy balance with direct input from measurements and experiments, projecting either final size based on a feeding level or projecting the amount of food needed (percentage of maximum consumption) to reach a preset size within a certain amount of time (Deslauriers *et al.*, 2017). On the other hand, the mechanistic or theoretical approach applies assumptions based on basic laws of thermodynamics and allometry and uses model parameters that can be hard to relate to real-world, empirical measurements (Nisbet *et al.*, 2012). Both these approaches require detailed, species-specific adjustments for model formulations, and neither is suited for simulating the dynamics of entire populations or including trophic interactions (Ohlberger *et al.*, 2011; Soudijn and de Roos, 2017).

**Figure 1:**
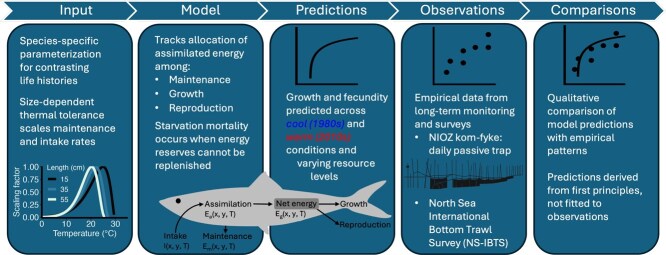
Schematic overview of modelling workflow, illustrating the sequence from model parameterization and formulation, through model predictions, to comparison with long-term empirical datasets, and evaluation of model performance.

Here, we present the analysis of physiological performance in fish, which is based on the analysis of a model that falls within the middle ground between the bioenergetics and theoretical approaches (Fig. 1). The model is rigorously based on mechanistic assumptions and scalings and uses a consistent energy balance equation for biomass production. At the same time, the model parameters are defined so that their values are informed by species-specific empirical findings and literature-reported estimates. Unlike physiological models that apply a uniform Arrhenius temperature scaling across life stages (Rose *et al.*, 2024), our framework accounts for intraspecific shifts in thermal sensitivity across body sizes, enabling more realistic predictions throughout ontogeny. By simulating the energetic balance and resulting somatic and reproductive growth at the individual level, and studying survival under fixed resource availability and fluctuating seasonal temperatures, we provide results that capture the complexities of how fish respond to environmental changes both via direct and indirect processes.

We applied the model to five fish species representing different functional and biogeographical guilds (Table 1). These species encompass temperate and boreal species, Atlantic cod (*Gadus morhua*) and Atlantic herring (*Clupea harengus*), as well as temperate and subtropical species, European sprat (*Sprattus sprattus*) and thinlip mullet (*Chelon ramada*), and a resident coastal species, the five-bearded rockling (*Ciliata mustela*). The predicted model outcomes were placed alongside completely independent findings from survey data on the same species in the southern North Sea coastal ecosystem (Table 2) *a posteriori*, without any calibrations to the model to fit the observations (Fig. 1). For this comparison, we focused on two periods with cooler (1980s) and warmer (2010s) seawater temperature conditions. Like many marine systems, this study region has experienced strong warming. We therefore tested whether our model outcomes under the contrasting temperature regimes of the 1980s (cooler) and 2010s (warmer) revealed differences in fish growth, survival and reproductive output consistent with empirical observations from these periods.

**Table 1:** Species parameterized in the generic physiological model including their guilds and characteristic life-history stage in the Wadden Sea

Species	Common name	Biogeographic guild	Functional guild	Wadden Sea life stage
*G. morhua*	Atlantic cod	Boreal^a^	Marine juvenile^c^	Juvenile
*C. harengus*	Atlantic herring	Boreal	Marine juvenile Marine seasonal migrant^d^ Marine adventitious^e^	Juvenile–adult
*C. mustela*	Five bearded rockling	Boreal	Estuarine resident^f^	Juvenile–adult
*S. sprattus*	European sprat	Lusitanian^b^	Marine seasonal migrant Marine adventitious	Juvenile–adult
*C. ramada*	Thinlip mullet	Lusitanian	Opportunist^g^	Juvenile–adult

Biogeographic guilds follow Jiming (1982), while functional guilds are defined according to Elliott and Hemingway (2002) and Elliott *et al.* (2007).

^a^Distribution centred north of the English Channel (cold-water affinity).

^b^Distribution centred south of the English Channel (warm-water affinity).

^c^Regularly enter estuaries but use nearshore marine waters as alternative habitat.

^d^Enter estuaries in large numbers as juveniles.

^e^Enter estuaries during certain times of the year.

^f^Occasionally (irregularly) enter estuaries, most frequently occur in high salinity (~35 PSU), lower reaches and coastal marine waters.

^g^Complete (or capable of) entire life cycle within an estuarine environment.

**Table 2:** Empirical sources used to corroborate predictions regarding body size and condition from the physiological model

Species	Variable in data	Method	Location	Time period	Source
*G. morhua*	Length-at-age	International Bottom Trawl Survey (NS-IBTS)	North Sea	1980s–2010s	ICES, 2020, 2023^a^
	Condition	Landings and research vessel data	Dutch North Sea	1968–1972	Daan, 1974 ^b^
		International Bottom Trawl Survey (NS-IBTS)	North Sea	2010s	ICES, 2020, 2023^a^
*C. harengus*	Length-at-age	International Bottom Trawl Survey (NS-IBTS)	North Sea	1980s–2010s	ICES, 2020, 2023^a^
	Length distribution	Passive fish trap	Wadden Sea	1980s–2010s	van Leeuwen *et al.*, 2023
*C. mustela*	Length-at-age	Passive fish trap	Wadden Sea	1974–1978 1998	Śmietana, 1992; van Leeuwen *et al.*, 2023
	Length distribution	Passive fish trap	Wadden Sea	1980s 2010s	van Leeuwen *et al.*, 2023
*S. sprattus*	Length-at-age	International Bottom Trawl Survey (NS-IBTS)	North Sea	1980s 2010s	ICES, 2020, 2023^a^
*C. ramada* ^c^	Length-at-age	Passive fish trap Survey^d^	Wadden Sea	1973–1984 2005–2015	Bolle *et al.*, 2021; van Leeuwen *et al.*, 2023

Please see Results section for more details.

^a^Downloaded from https://datras.ices.dk/ on April 25, 2023. Data include all available quarters: 1980s (Q1), 2010s (Q1–Q3).

^b^Species-specific field data points collected using WebPlotDigitizer (Rohatgi, 2022).

^c^Please note that data from the closely related species, *Chelon labrosus*, was used to represent our study species, *C. ramada.*

^d^Additional surveys (2009 Wadden Sea; 2001 to present Afsluitdijk) to supplement underrepresented length and age classes.

## Materials and Methods

We studied the additive effects of food and temperature dependence on physiology and performance in fish (Fig. 1). We applied the individual-level model definition used widely in physiologically structured population models (PSPMs, see de Roos and Persson, 2013). This foundation captures a mechanistic energy balance and process rates determined by the individual's body size, food availability and temperature. We relate the model to empirical measurements at two points: First, the species-specific parameterization relies on literature-reported findings for size scalings of metabolic rate and food intake rate, for example. Second, following analysis, the model results are confronted with completely independent species-specific findings from research or fisheries surveys or monitoring data. We avoid model fitting and rather compare the model predictions for body size and condition qualitatively with what has been observed in the ecosystem for the five study species.

### Model description

#### Temperature-dependent physiological model

We used standard components for the base of our model formulation, with physiological processes defined at the individual level and dependent on available resource or energy as originally defined for the consumer-resource PSPM in Persson *et al.* (1998). These physiological functions were adapted to include temperature-dependent scaling as developed in Karås and Thoresson (1992) and Ohlberger *et al.* (2011). Previous modelling approaches that account for temperature and resource dependence included environmental temperature as a constant throughout the year (Ohlberger *et al.*, 2011) or using a stepwise temperature change between winter and summer conditions (van de Wolfshaar *et al.*, 2008); in contrast, we added a seasonally varying temperature (with a sigmoidal function) as the environment. These three elements—physiology dependent on resource, physiology dependent on temperature and seasonally varying temperature—constitute the full base model that we applied to five marine fish species. We derived the parameterizations for five fish species from the literature (Table 3); the details on derivation and temperature-dependent parameterization procedure for each species can be found in the supplementary files (S1–S3).

**Table 3:** Values of the model parameters for the five species examined in this study

	Value							
Symbol	Cod	Herring	Rockling	Sprat	Mullet	Unit	Description	Source
Ontogeny								
${w}_{\mathrm{b}}$	0.00039	0.0007	0.00149	0.0005	0.00038	g	Egg mass	1, 2, 3, 2, 4
$q$ j	0.7	0.7	0.8	0.9	0.8	–	Juvenile maximum condition	1, 2, 5, 2, 6
$q$ a	1.2	1.13	1.03	1.4	1.0	–	Adult maximum condition	7, 2, 8, 2, 9
$q$ r	0.7	0.7	0.8	0.9	0.8	–	Threshold condition for spawning	1, 2, 5, 2, 6
*k*r	0.5	0.5	0.5	0.5	0.5	–	Gonad to offspring conversion efficiency	1, 2, 10, 2, 10
*E*_d_	15	17	11	17	2	day	Duration of egg period	1, 2, 11, 12, 13, 14
*A*_f_	22	25	16	25	7	day	Age at first feeding	1, 2, 11, 15, 16, 12, 13, 14
*L*_m_	62	14	14	9	25.9	cm	Maturation length	7, 2, 17, 18, 19, 2, 20
${\lambda}_1$	4.95	5.65	5.26	5.4	4.48	cm ${\mathrm{g}}^{-{\mathrm{\lambda}}_2}$	Allometric size scalar	20, 2, 21, 22, 2, 20
${\lambda}_2$	0.325	0.32	0.3174	0.33	0.341	–	Allometric size exponent	20, 2, 21, 22, 2, 20
Consumption								
*d*_1_	0.2	0.97	1.80	0.46	0.3	$\mathrm{day}\ {\mathrm{g}}^{-\left(1+{\mathrm{d}}_2\right)}$	Allometric scalar	23, 23, 23, 23, 23
*d*_2_	0.635	0.303	0.05	0.215	0.52	–	Allometric exponent	23, 23, 23, 23, 23
${\vartheta}_{\mathrm{a}}$	2.2	1.8	2.0	2.2	1.81	–	Allometric scalar	24, 25, 26, 23, 27, 28
${\theta}_{\mathrm{a}}$	0.075	0.0775	0.075	0.0775	0.075	–	Allometric exponent	23, 27, 23, 27, 23
${\gamma}_{\mathrm{a},\max }$	22	23	27	25	37	${\mathrm{g}}^{-{\mathrm{v}}_{\mathrm{a},\max }{}^{\circ}}\mathrm{C}$	Allometric scalar	29, 30, 32, 33, 34, 35, 36, 37, 38, 33, 39
${v}_{\mathrm{a},\max }$	−0.0435	−0.0475	−0.0275	−0.0475	−0.031	–	Allometric exponent	23, 40, 23, 23, 23
${\gamma}_{\mathrm{a},\mathrm{opt}}$	16.78	17	16	21	30	${\mathrm{g}}^{-{\mathrm{v}}_{\mathrm{a},\mathrm{opt}}{}^{\circ}}\mathrm{C}$	Allometric scalar	30, 31, 41, 42, 33, 34, 35, 36, 37, 38, 28, 39, 42, 43
${v}_{\mathrm{a},\mathrm{opt}}$	−0.078	−0.031	−0.072	−0.055	−0.041	–	Allometric exponent	23, 41, 42, 23, 23, 23
Metabolism								
$\rho$ _1_	0.03	0.03	0.03	0.03	0.03	${\mathrm{g}}^{\left(1-\mathrm{\rho}^2\right)}\,\mathrm{day}^{-1}$	Allometric scalar	1, 2, 23, 2, 23
$\rho$ _2_	0.8	0.8	0.79	0.8	0.79	–	Allometric exponent	1, 2, 44, 45, 2, 44, 45
*k*_e_	0.5	0.5	0.5	0.5	0.5	–	Resource energy conversion efficiency	10, 44, 2, 10, 44, 2, 10, 44, 46, 47, 48
${\vartheta}_{\mathrm{m}}$	2.6	2.2	2	2.25	2.27	–	Allometric scalar	49, 42, 45, 50, 51, 52, 6
${\theta}_{\mathrm{m}}$	0.077	0.081	0.077	0.081	0.077	–	Allometric exponent	49, 52, 23, 52, 23
${\gamma}_{\mathrm{m},\max }$	24	24	29	26.5	42	${\mathrm{g}}^{-{\mathrm{v}}_{\mathrm{m},\max }{}^{\circ}}\mathrm{C}$	Allometric scalar	49, 53, 54, 55, 32, 42, 23, 27, 56, 57
${v}_{\mathrm{m},\max }$	−0.029	−0.05	−0.029	−0.05	−0.0295	–	Allometric exponent	23, 41, 42, 23, 27, 23
${\gamma}_{\mathrm{m},\mathrm{opt}}$	15.5	16.5	17.5	20	34	${\mathrm{g}}^{-{\mathrm{v}}_{\mathrm{m},\mathrm{opt}}{}^{\circ}}\mathrm{C}$	Allometric scalar	53, 58, 41, 42, 23, 27, 56, 59
${v}_{\mathrm{m},\mathrm{opt}}$	−0.02	−0.028	−0.025	−0.028	−0.04	–	Allometric exponent	23, 41, 42, 23, 23, 23

(1) van Leeuwen (2012). (2) Huss *et al.* (2013). (3) Dye (2021). (4) Kooijman (2020). (5) Harris and Hislop (1978). (6) Flowerdew and Grove (1980). (7) Soudijn *et al.* (2020). (8) Badsha (1977). (9) Koutrakis (2011). (10) Persson *et al.* (1998). (11) Dando (1975). (12) Huss *et al.* (2012). (13) Mousa (2010). (14) Crosetti and Blaber (2015). (15) Russell (1976). (16) Brook (1888). (17) Badsha and Sainsbury (1978). (18) Cohen *et al.* (1990). (19) Munk and Nielsen (2005). (20) Fishbase (Froese and Pauly (2024). (21) Silva *et al.* (2013). (22) Wilhelms (2013). (23) Best fit by authors. (24) Imsland *et al.* (2005). (25) McGurk (1984). (26) Mazumder *et al.* (2015). (27) Bernreuther *et al.* (2009). (28) Okamoto *et al.* (2006). (29) Björnsson *et al.* (2001). (30) Björnsson *et al.* (2007). (31) Peck *et al.* (2003). (32) Blaxter (1960). (33) Jobling (1981). (34) Dahlke *et al.* (2020). (35) Dye *et al.* (2024). (36) Peck *et al.* (2012). (37) Baumann *et al.* (2006). (38) Peck *et al.* (2013). (39) Dye *et al.* (2025). (40) Brawn (1960). (41) Bernreuther *et al.* (2013). (42) Moyano *et al.* (2020). (43) Peterson *et al.* (2000). (44) Peters (1986). (45) Clarke and Johnston (1999). (46) Hickling (1970). (47) Payne (1976). (48) Cardona (1999). (49) Tirsgaard *et al.* (2015). (50) Fry (1947). (51) Eliason and Anttila (2017). (52) Meskendahl *et al.* (2010). (53) Freitas *et al.* (2010). (54) Zanuzzo *et al.* (2019). (55) Norin *et al.* (2019). (56) Cucco *et al.* (2012). (57) Madeira *et al.* (2012). (58) Soofiani and Hawkins (1982). (59) Moore (1976). ‘Best fit by authors’ indicates parameters fitted by expert judgement when experimental results varied across studies or conditions.

As is customary in PSPMs, the physiological state of individuals was determined by two types of mass: the structural mass (*x*) consisting of bones and vital organs that cannot be starved away in resource poor environments and the reserve mass (*y*) consisting of fat, muscle and gonadal tissues. The total body mass of an individual was equal to the sum of structural and reserve masses (*x* + *y*), while the ratio of the two (*y*/*x*) represents the body condition. The model metric of body condition differs from Fulton’s condition index [= 100 × (weight/length^3^)], which is based on total weight and length. For consistency with field data, all condition results (Section 3.2) are presented using Fulton’s condition index. Standard mass is defined as the sum of structural and reserve mass at maximum level, excluding gonad mass (Table 4). Unlike structural mass, reserve mass could be allocated to cover maintenance costs [*E*_m_(*m*)] when these exceeded acquired energy [*E*_a_(*m*)].

**Table 4:** Individual-level model variables and equations

Subject	Equations
Standardized mass (g)	$m(x)=x\left(1+{q}_j\right)$
Total mass (g)	$w(x)=x+y$
Body length (cm)	$L(x)={\lambda}_1m{(x)}^{\lambda_2}$
Intake (g day^−1^)	$I\left(x,y,T\right)={d}_1m{(x)}^{d_2}{r}_a\left(m(x),T\right){R}_l$
Juvenile: energy allocation to *x* when *L* < *L*_m_	$$ f\left(x,y\right)=\left\{\begin{array}{c}\frac{1}{\left(1+{q}_j\right){q}_j^2}{\left(\frac{y}{x}\right)}^2\kern0.5em if\ \frac{y}{x}<{q}_j\\{}\frac{1}{1+{q}_j}\kern3.75em \mathrm{otherwise}\end{array}\right.$$
Adult: energy allocation to *x* when *L* > *L*_m_	$$f\left(x,y\right)=\left\{\begin{array}{c}\frac{1}{\left(1+{q}_a\right){q}_a^2}{\left(\frac{y}{x}\right)}^2\kern0.5em if\ \frac{y}{x}<{q}_a\\{}\frac{1}{1+{q}_a}\kern3.75em \mathrm{otherwise}\end{array}\right.$$
Acquired energy (g day^−1^)	${E}_a\left(x,y,T\right)={k}_eI\left(x,y,T\right)$
Energy requirements for maintenance (g day^−1^)	${E}_m\left(x,y,T\right)={\rho}_1{\left(x+y\right)}^{\rho_2}{r}_m\left(m(x),T\right)$
Energy balance (g day^−1^)	${E}_g\left(x,y,T\right)={E}_a\left(x,y,T\right)-{E}_m\left(x,y,T\right)$
Fecundity (#)	$$F\left(x,y\right)=\left\{\begin{array}{ll}\frac{k_r\left(y-{q}_jx\right)}{w_b}\kern0.5em if\ L>{L}_m\ \mathrm{and}\ y>{q}_jx\\{}0\kern3.75em \mathrm{otherwise}\end{array}\right.$$
Temperature dependence	$r\left(x,y,T\right)=V{\left(x,y,T\right)}^{X\left(x,y,T\right)}{e}^{X\left(x,y,T\right)\left[1-V\left(x,y,T\right)\right]}$
	$V\left(x,y,T\right)=\frac{\left({T}_{\mathrm{max}}\left(x,y\right)-T\right)}{\left({T}_{\mathrm{max}}\left(x,y\right)-{T}_{\mathrm{opt}}\left(x,y\right)\right)}$
	$X\left(x,y,T\right)={W}^2{\left[1+{\left(1+\frac{40}{Y}\right)}^{0.5}\right]}^2\frac{1}{400}$
	$W=\left({T}_{\mathrm{max}}\left(x,y\right)-{T}_{\mathrm{opt}}\left(x,y\right)\right)\ \mathit{\ln}\ Q\left(x,y\right)$
	$Y=\left({T}_{\mathrm{max}}\left(x,y\right)-{T}_{\mathrm{opt}}\left(x,y\right)+2\right)\ \mathit{\ln}\ Q\left(x,y\right)$
	${T}_{\mathrm{opt}}\left(x,y\right)={\gamma}_{\mathrm{opt}}{\left(x+y\right)}^{v_{\mathrm{opt}}}$
	$T_{max}$ $\left(x,y\right)={\gamma}_{\mathrm{max}}{\left(x+y\right)}^{v_{\mathrm{max}}}$
	$Q\left(x,y\right)=\vartheta{\left(x+y\right)}^{\theta }$
Acquired energy (°C)	$T$ $_{a,\mathrm{opt}}\left(x,y\right)=\gamma_{a,\mathrm{opt}}$ ${\left(x+y\right)}^{v_{a,\mathrm{opt}}}$
	$T_{a,\mathrm{max}}$ $\left(x,y\right)=\gamma_{a,\mathrm{max}}$ $\left(x+y\right) v_{a,\mathrm{max}}$
	${Q}_a\left(x,y\right)={\vartheta}_a{\left(x+y\right)}^{\theta_a}$
Metabolism (°C)	$T_{m,\mathrm{opt}}$ $\left(x,y\right)=\gamma_{m,\mathrm{opt}}$ $\left(x+y\right) v_{m,\mathrm{opt}}$
	$T_{m,\mathrm{max}}\left(x,y\right)=\gamma_{m,\mathrm{max}}{\left(x+y\right)}^{v_{m,\mathrm{max}}}$
	${Q}_m\left(x,y\right)={\vartheta}_m{\left(x+y\right)}^{\theta_m}$
State variable dynamics	
Growth in structural mass, *x*	$$\frac{dx}{dt}=\left\{\begin{array}{c}f\left(x,y\right){E}_g\left(x,y,T\right)\kern3.00em if\ {E}_g>0\\{}0\kern9.25em \mathrm{otherwise}\end{array}\right.$$
Growth in reserve mass, *y*	$$\frac{dy}{dt}=\left\{\begin{array}{c}\left(1-f\left(x,y\right)\right){E}_g\left(x,y,T\right)\kern3.00em if\ {E}_g>0\\{}{E}_g\left(x,y,T\right)\kern8em \mathrm{otherwise}\end{array}\right.$$

An individual acquired energy through resource intake [*I*(*m*)], an allometric function (Table 4—Intake; S1-1) depending on the standardized mass and accounting for the temperature-dependent term (*r*_a_) and a constant resource level (${\mathrm{R}}_{\mathrm{l}}$; Fig. 1). For resource intake, we used a feeding level instead of a dynamically linked resource density. Conceptually, feeding level can range between 0 and 1, with 0 corresponding to starvation (i.e. no intake) and 1 representing the amount of resource required for maximum growth (Chaparro-Pedraza and de Roos, 2019). In our simulations, we applied three discrete feeding levels representing low, moderate and high resource availability (Section 2.3).

Following allocation rules in Persson *et al.* (1998), assimilated energy [*E*_a_(m)] from the resource first covered maintenance costs [*E*_m_(m)] and, if an energy surplus remains (*E*_g_(m) > 0), energy was then used for growth in reserve and structural mass. If assimilated energy was not sufficient to cover maintenance costs (*E*_g_(m) < 0), starvation occurred and reserve mass was used to cover maintenance costs. During periods of starvation, energy allocation prioritized the recovery of body condition over structural mass growth, as reflected by the quadratic term in the energy allocation formula (Table A2 in Huss *et al.*, 2012).

Fish reach reproductive maturity at the maturation length (*L*_m_), where individuals transition from juvenile to adult life stages. The maximum ratio of reserves to structural mass was given by *q*_j_ and *q*_a_ for juveniles and adults, respectively, parameters that were species specific and derived from the literature. The maximum reserve to structural mass ratio was higher in adults than in juveniles, representing additional investment in reproductive tissues (*q*_a_ vs. *q*_j_—Table 3; S1-2). If reserve mass (*y*) was less than or equal to *q*_j_*x*, reproduction did not occur. Reproduction occurred on the first day of the model year, thereby generalizing across reproductive strategies (e.g. capital vs. income breeding). In the model, the fish is represented as a female, as females invest substantially more energy into reproduction compared to males. Consequently, all accumulated gonadal mass on the first day of the model year was converted into the number of eggs, *F*(*x*, *y*), described by the fecundity equation (Table 4), which accounts for the reproduction efficiency (*k*_r_) (i.e. gonad-offspring conversion) and the species-specific egg mass (*w*_b_).

#### Temperature dependence

Temperature-dependent rates of maintenance metabolism and food intake were implemented by multiplying each rate by a temperature dependent factor (${\mathrm{r}}_{\mathrm{a}},{\mathrm{r}}_{\mathrm{m}}$—Table 3 and Fig. 1, following Ohlberger *et al.*, 2011). The temperature adjustment factors are formulated for each physiological process accounting for size-specific temperature tolerance (lethal) ranges and optimum temperatures (Karås and Thoresson, 1992; Ohlberger *et al.*, 2011). This method addresses criticisms of standard Arrhenius functions, which describe the exponential dependence of chemical reaction rates on temperature, when extrapolating the temperature-dependence above thermal optimal (Englund *et al.*, 2011; Rose *et al.*, 2024). While extended Arrhenius formulations (e.g. Kooijman, 2010; Dambrine *et al.*, 2020) incorporate species-level thermal limits, they do not explicitly account for differences in thermal physiology across different body sizes and life stages (Dahlke *et al.*, 2020).

Parameters were estimated and fitted from data on species-specific temperature dependence (Table 3). Data from various experimental approaches were utilized for the intake scaling based on the correlation between long-term temperature preference(s), optimum growth temperature(s) and lethal temperature(s) (Jobling, 1981; Pörtner and Peck, 2010; S1, 3-4), while respiration and activity experiments provided the necessary data for the maintenance scaling (Karås and Thoresson, 1992). Species generally experience temperature-driven constraints on oxygen supply, commonly described as aerobic scope (Pörtner and Knust, 2007). In our framework, this concept is represented through parametrization of the temperature-dependent maintenance costs, which are based on size-dependent relationships and species-specific empirical data on respiration and activity (Supplemental Section 3). Although oxygen dynamics are not explicitly modelled, their temperature-mediated effects on fish performance are implicitly incorporated into the model. A more detailed description of the parametrization estimation and fitting procedures are provided in the species-specific sections (S1, 3-4).

#### Species selection and parameterization

We applied the model to five different species that offer logical contrasts in thermal physiologies and life history strategies. The choice of the species was also inspired by the species assemblage of the Wadden Sea (southern North Sea), a fish community that has been well studied in terms of changes in its composition (Cardoso *et al.*, 2015; van Walraven *et al.*, 2017; Chust *et al.*, 2024), abundance and biomass (van der Veer *et al.*, 2015; Tulp *et al.*, 2017; van der Veer *et al.*, 2022). Importantly, historical changes in resource productivity (Philippart *et al.*, 2007; van Beusekom *et al.*, 2019), as well as long-term, high-resolution seawater temperature measurements (van Aken, 2003; van Leeuwen *et al.*, 2021, 2022), are available for this system.

Two components of the model were informed by data: (1) species-specific parameter values for vital rates (see below in Section 2.1.1 and in Table 3), and (2) the model equation representing the seasonal variation in seawater temperature (described below in Section 2.2). The dynamic model outcomes were an emergent property and were not directly fitted to empirical data.

### Data

#### Water temperature

**Figure 2:**
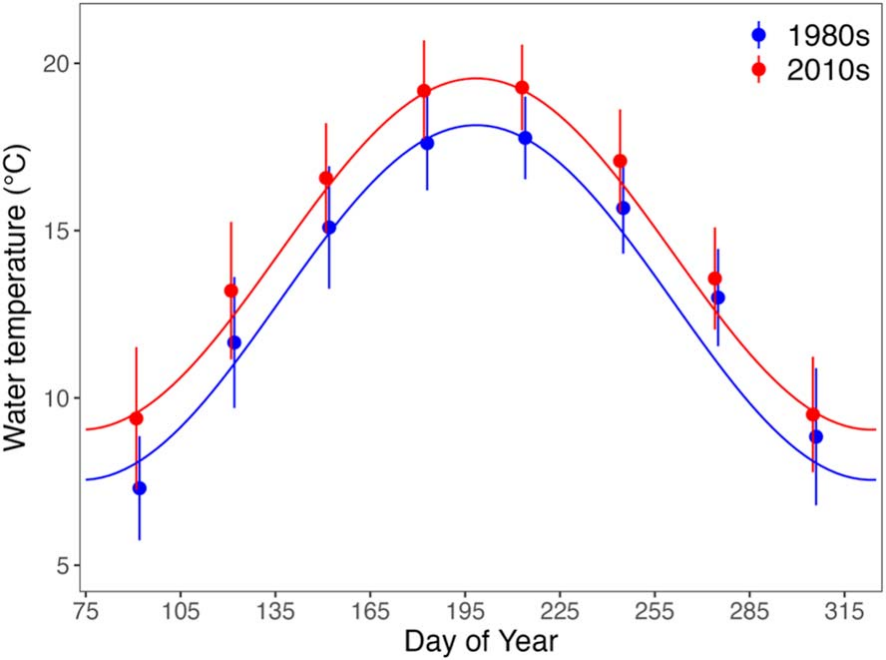
Monthly (mean ± SD) water temperatures during contrasting periods (1980s and 2010s) at a fixed location in the western Wadden Sea (53.002 N, 4.789 E) and the sinusoidal curves fitted to the data representing the 250-day growth season utilized for model simulations.

To account for seasonally varying seawater temperature, a sinusoidal curve was included in the model formulation (Pethybridge *et al.*, 2013). Curves were fitted to monthly mean temperatures from the Royal Netherlands Institute for Sea Research (NIOZ) jetty (van Leeuwen *et al.*, 2021) for two different, 10-year periods (Fig. 2): 1980–1989 and 2010–2019, representing temperature curves for ‘a long-term average’ (1980s) and ‘a hot’ (2010s) climatological period in the Wadden Sea. The mean annual water temperature (${T}_{\mathrm{mean}}$), amplitude (a), phase shift of sinusoid ($\mathrm{\omega}$) and growing season (GS) parameters were used in the water temperature equation (Table 5) to empirically fit a sinusoidal function to the *in situ* measurements through visual estimation. The individual-level model used daily water temperatures obtained from the sinusoidal function.

**Table 5:** Environmental data and the temperature equation applied in model simulations

Symbol	Value		Unit	Description	Source
	1980s	2010s			
*T*_mean_	12.85	14.3	°C	Annual water temperature	Derived from Wadden Sea measurements; van Leeuwen *et al.*, 2021
*a*	5.3	5.25	–	Amplitude	Derived; van Leeuwen *et al.*, 2021
*ω*	187	187	–	Phase shift of sinusoid	Derived from Wadden Sea growing season
*R*_l_	1, 0.7 or 0.5			Constant resource level	
GS	250		days	Length of growing season	
		
Water temperature		$T\left(\mathrm{t}\right)={T}_{\mathrm{mean}}+a\ast \sin \left(\frac{2\mathrm{\pi} \left(t+\mathrm{\omega} \right)}{\mathrm{GS}}\right)$			

#### Link with empirical findings

Field surveys and monitoring programmes yielded species-specific data to confront model outcomes for body size and condition (Table 2 and Fig. 1). Observations were generally collected from the ‘NIOZ kom-fyke’, a long-term, daily monitored, passive fish trap (fyke; mesh size, 20 mm) located near the entrance of Dutch Wadden Sea (Table 2). Kom-fyke catches correspond, on-average, to a constant sampling fraction of most western Wadden Sea fish species, in particular the five species used to test the model (van der Meer *et al.*, 1995; Table 1). Data from the North Sea International Bottom Trawl Survey (NS-IBTS; Table 2) filled gaps in the length-at-age data from Wadden Sea catches. The size distributions of herring and sprat in the North Sea and Wadden Sea were assumed to be the same since these mobile species frequently enter and exit coastal systems, particularly early in life (Couperus *et al.*, 2016; Maathuis *et al.*, 2023; Table 1). The NS-IBTS is a broad survey spanning the North Sea using standardized trawl gear with a 20-mm codend. We leveraged existing data to compare model outcomes; however, comparable data from field systems (be it from surveys or monitoring) were not available for every species and model variable. In the Results section, model output is presented according to the available data source (Table 2).

### Simulations

Seasonal changes in water temperatures were simulated under three constant resource levels (0.5, 0.7 and 1.0) in two temperature scenarios: cooler, which is representative of and based on SST measured in the 1980s; and warmer, which is representative of and based on SST measured in the 2010s. These six simulations were conducted for each of the five fish species. A resource level of 1.0 represents sufficient resources for maximum fish growth, while levels of 0.7 and 0.5 correspond to moderate and low resource availability, respectively. Constant resource levels were used to isolate temperature and life-history effects and to reduce uncertainty arising from poorly constrained resource dynamics (see Section 4.6 for further justification).

The model simulations were performed using the Escalator Boxcar Train-tool (EBTtool) software package (Version Mar 21 2020; de Roos, 2023). The EBTtool is a numerical method designed to integrate equations of physiologically structured models (de Roos and Persson, 2013) following the EBT framework (de Roos, 1988). The simulations used a daily time step and ran for the duration of the species’ maximum reported lifespan (Froese and Pauly, 2024). Each simulation began from the species’ birth weight, as specified in Table 3, and continued until either the species-specific maximum lifespan was reached or starvation mortality occurred. Each model year was 250 days (Fig. 2) and represented the Wadden Sea growing season (Table 5). The other 115 days were excluded since physiological processes were assumed to slow down during this part of the year, and prior studies have found qualitative identical outcomes independent of season duration (Persson *et al.*, 1998).

## Results

The interactive effects of temperature and food dependence were species specific for the simulated body sizes, body condition and fecundity. We discuss the model predictions below and compare these qualitatively with empirical observations from the field. The outcomes are organized by output variable and specified for patterns at the species levels.

### Length-at-age

**Figure 3:**
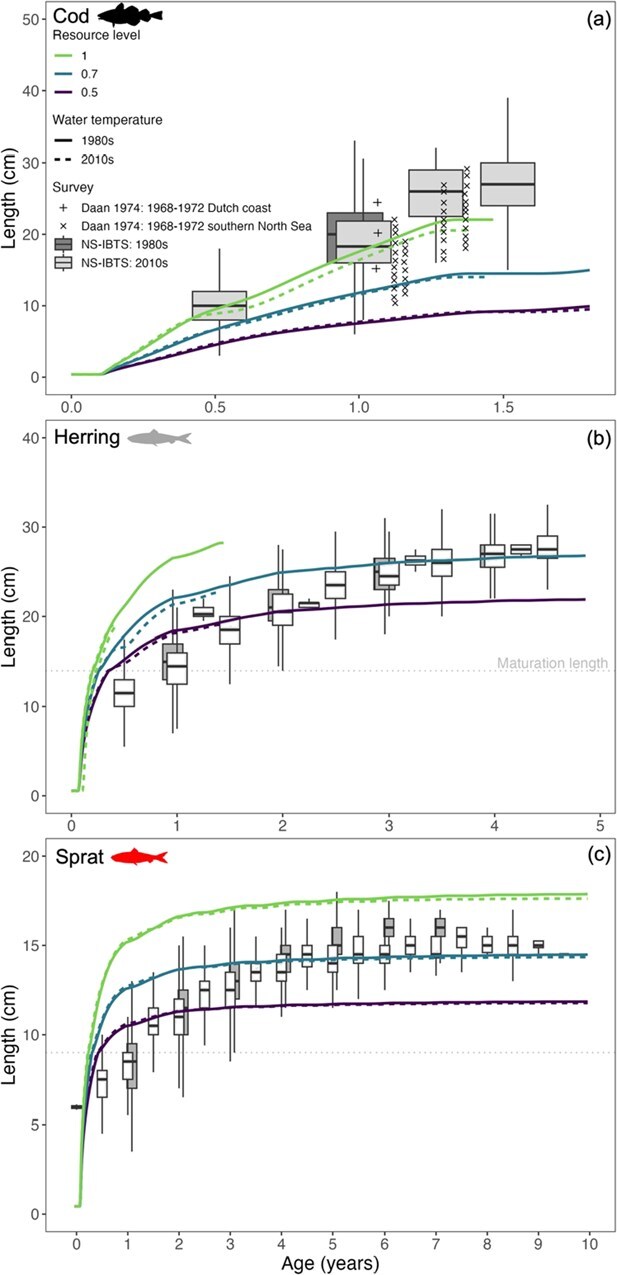
Model-predicted and field measurements of length-at-age for Atlantic cod (panel a), Atlantic herring (panel b) and European sprat (panel c). Model predictions included three resource levels and two temperature regimes. Details on the field data are provided in Table 2. Please note the difference in scaling on the axes. The maturation length (grey dotted line) is shown for herring and sprat but not for cod. Predicted length in cod did not reach length-at-maturation.

**Figure 4:**
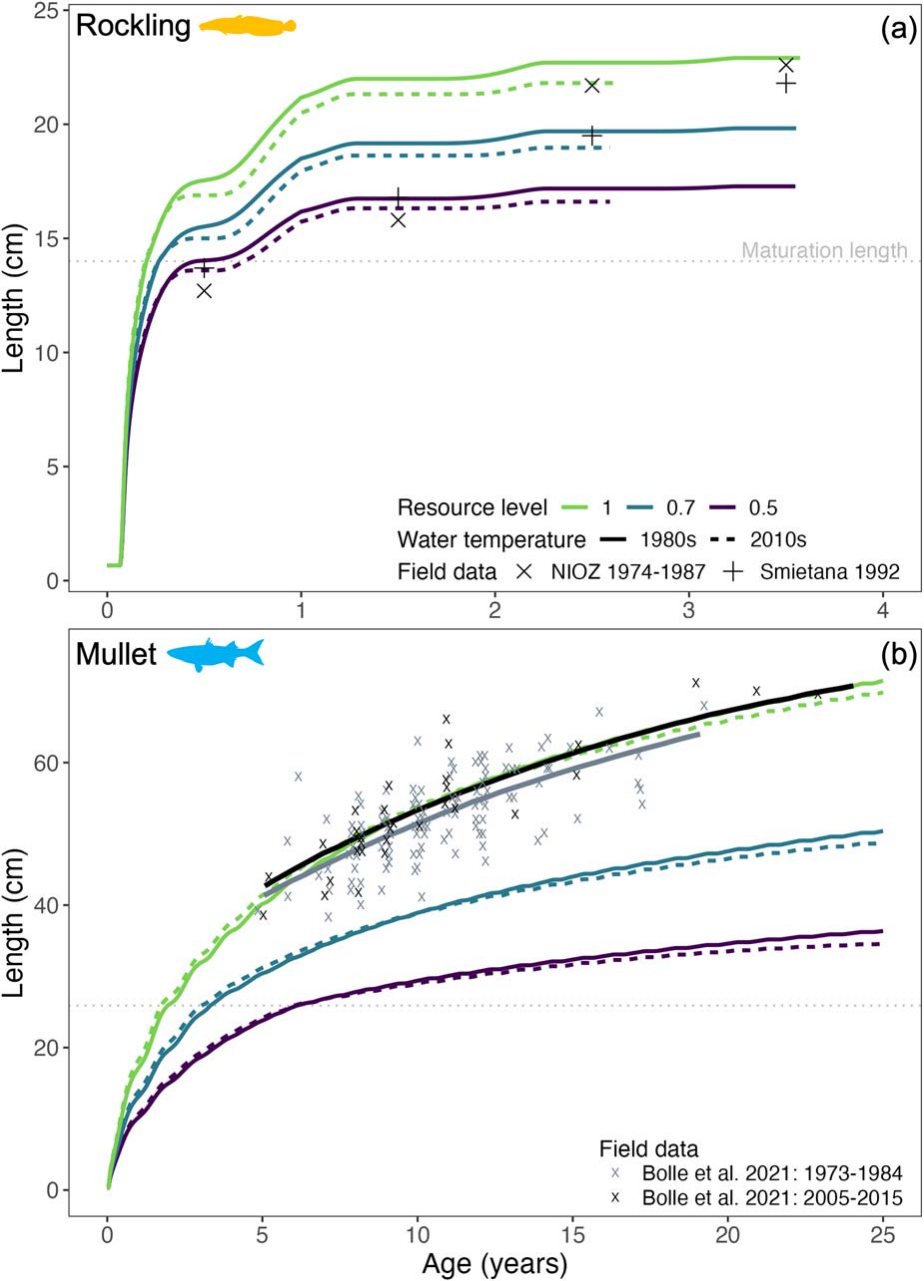
Model-predicted length-at-age for five bearded rockling (panel a) and thinlip mullet (panel b) under contrasting environmental conditions compared to western Wadden Sea field data (Table 2). Mullet: lines are best fitted to data. Please note the difference in scaling on the axes.

For all five species simulations, length-at-age decreased with decreasing resource level: feeding level 1.0 resulted in fast growth up to the maximum sizes reached in the simulations, while feeding levels of 0.7 and 0.5 resulted in growth curves with the same shapes, but reaching smaller sizes (Figs 3 and 4). The growth curves at three feeding levels showed large and monotonic differences for all species. The impact of temperature was inconsistent between species, with cod and herring being most susceptible to negative impacts of higher temperature. The growth curves from simulations with cool and warm temperature were overall close together, but survival (of cod and herring) and fecundity (of mullet and sprat) were affected, which is discussed in detail below. The model accurately predicted length-at-age and maximum lengths of sprat and herring in later life stages but overestimated length-at-age in early life (i.e. at age 0 to 1; Fig. 3b and c). The variation in growth curves due to feeding level was in the same range as variation of observed length-at-age in these species, as well as in rockling. For mullet, only the highest feeding level simulations resulted in a length-at-age curve that was in the range of observed findings. Mullet and rockling experienced a ~ 1- to 2-cm reduction in maximum lengths reached for the warm (2010s) simulations compared to the cool simulations (1980s) (Fig. 4), an effect present at all three feeding levels.

**Figure 5:**
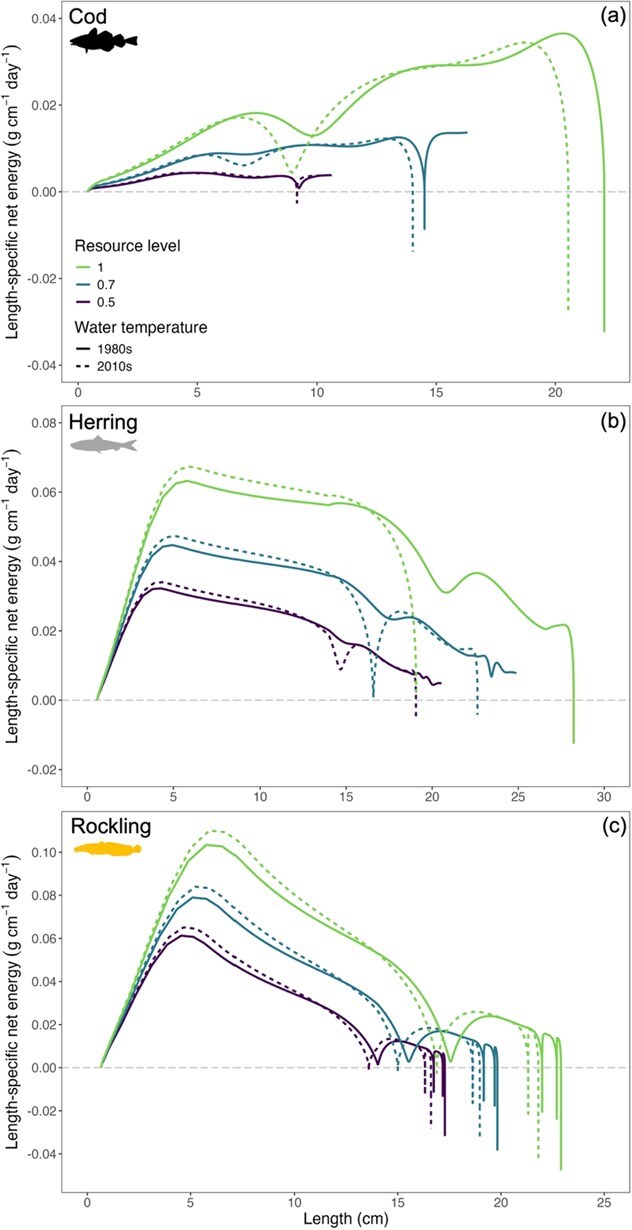
Model-predicted length-specific net energy (g cm^−1^ day^−1^) for Atlantic cod (panel a), Atlantic herring (panel b) and five bearded rockling (panel c) under contrasting environmental conditions. The grey dashed line indicates zero net energy, serving as the threshold between energy gain and loss. Please note the difference in scaling on the axes.

Growth in length for cod, herring and rockling ceased after a negative energy balance was reached and reserve mass (Fig. 5) was exhausted. In cod and herring, larger body sizes combined with lower thermal thresholds limited the ability to restore depleted energy reserves, prevented maintenance requirements from being met and resulted in mortality at 1.25 and 0.5 years, respectively (Fig. 3a and b). In rockling, seasonal changes in length-at-age highlighted trade-offs in energy allocation and growth at suboptimally warm temperature. Regardless of resource level and temperature regime, rockling ceased to grow midway through their first year and grew less in the second year (Fig. 4a). The growth cessation was less pronounced in their second year because rockling were reproductively mature and a portion of the surplus energy was allocated towards developing reproductive tissue. Simulated temperatures exceeded the size-specific thermal optimum of rockling, causing a negative energy balance and growth cessation during the summer peak of the growing season (Fig. 2). However, once temperatures decreased in the autumn, rockling could once again utilize reserve energy for somatic and reproductive growth instead of allocating all reserves to cover maintenance costs (Fig. 5c). Furthermore, growth of rockling ceased ~1 year earlier during the 2010s simulations with higher temperatures (Fig. 4a).

### Condition

**Figure 6:**
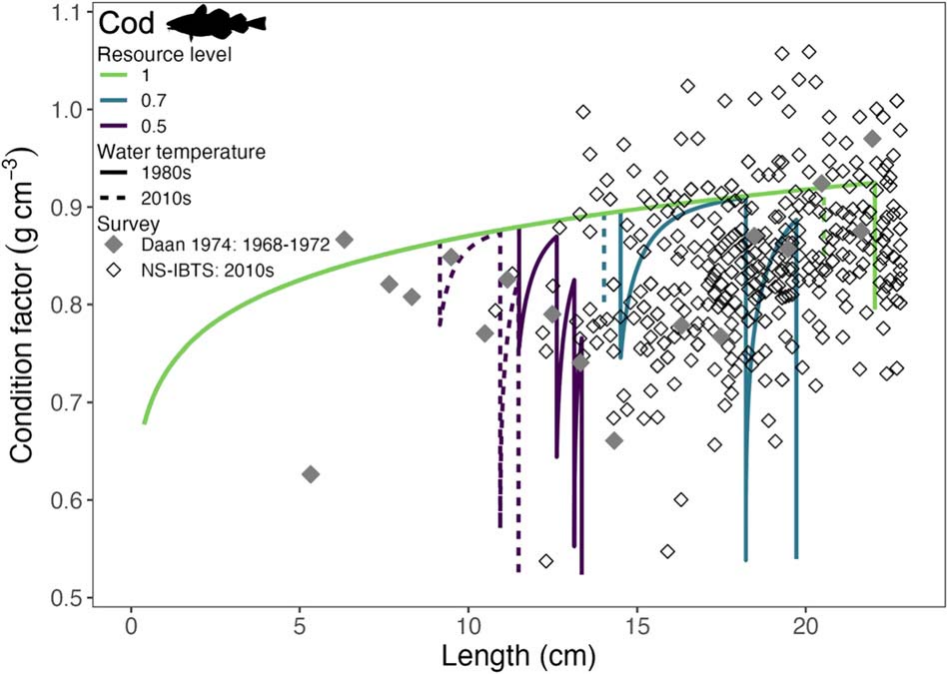
Simulated length-specific condition factor of Atlantic cod compared to field data [NS-IBTS (1980s Q1 and 2010s Q1 and Q3), 1-mm-length class reporting unit; Table 2], with both datasets expressed as Fulton’s condition index.

The simulation captured the observed variability in length-specific somatic condition of cod, with both empirical and simulated values quantified using Fulton’s condition index. Cod condition increased with length in both temperature simulations and agreed well with data from southern North Sea cod landings data during 1968–1972 and the 2010s North Sea survey data (Fig. 6 and Table 2). The resource level(s) encountered by cod in the field are unknown, and this complicates disentangling how resource level impacted observed changes in body condition of fish of different lengths. Our results, however, captured most of the variation within the field data including the clustered pattern of field measurements (i.e. condition factor of 0.7–0.9 at 10- to 20-cm lengths), enclosing the lowest condition values, yet underpredicting the highest observations (i.e. condition factor > 1 at 10- to 20-cm lengths, Fig. 6).

### Length distribution

**Figure 7:**
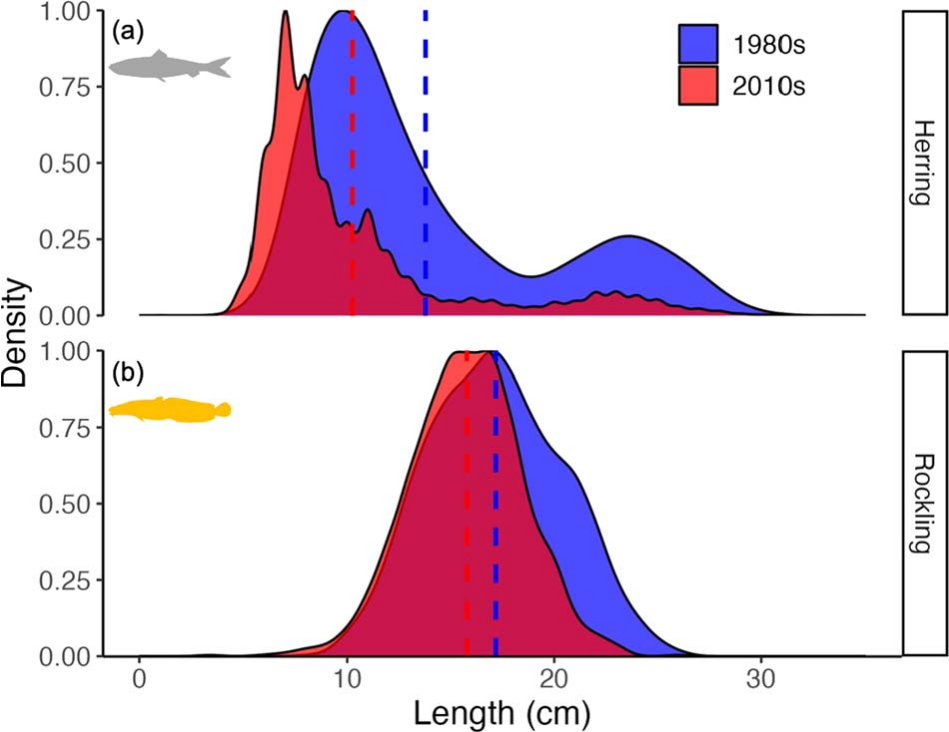
Comparison between 1980s and 2010s length distribution of Atlantic herring (panel a) and five bearded rockling (panel b) from western Wadden Sea field data (Table 2). Mean decadal lengths are indicated by dashed lines, and the maximum density is scaled to 1. Sample sizes: Rockling 1980s (*n* = 2738), 2010s (*n* = 1609); Herring 1980s (*n* = 381 950), 2010s (*n* = 131 218).

Modelled growth trajectories for herring and rockling were consistent with the observed reductions in length distributions from the western Wadden Sea. In the field data, the mean herring length distribution (Table 2) was reduced by 3.6 cm between the two time periods simulated in this study (Fig. 7a). In the cooler 1980s, 30% more herring was found at lengths greater than 10.2 cm, which was the mean length in the warmer 2010s. Herring grew to a maximum length of ~20 cm in the 2010s simulations (Fig. 3b). These model predictions are corroborated because <10% of herring lengths in the 2010s were > 20 cm. Furthermore, the maximum herring length predicted in the cooler 1980s at unlimited resources (28.2 cm, Fig. 3b) corresponds well to the largest herring measured during that time (Fig. 7a). For rockling, the observed length distributions were reduced by 1.4 cm in the warmer 2010s compared to the cooler 1980s (Fig. 7b). The simulations captured this reduction (Fig. 4a) although to a lesser extent than the field data as only a ~1 cm reduction was predicted by the model.

### Fecundity

Lifetime fecundity predictions were species specific and depended on the resource level. As a result of failing to reach maturation length (Table 3 and Fig. 3a), cod did not reproduce in any of the simulations, which is not an unexpected result given that cod uses the Wadden Sea as juvenile only (Table 1). At maximum resource level and warmer (2010s) water temperatures, rapid increases in size for herring in the first year of life led to an exhaustion of energy reserves and no survival after first reproduction (Fig. 5b). The individuals that persisted through the cooler 1980s simulations (at intermediate and low resource levels, Fig. 3b) reproduced for six consecutive years. Counterintuitively, lower resource levels enhanced herring survival (allowing survival >1 year old), but only for the colder (1980s) temperatures. The maximum fecundity of herring under moderate (0.7) and low (0.5) resource levels was 65 000 and 34 000 eggs, respectively (S1-5, Fig. 2). Rockling experienced a single reproductive event after the first year of life in both simulation periods, yielding ~2000 to 6000 eggs at 0.5 and 1.0 resource levels, respectively. During the cooler 1980s, rockling exhibited a 2.4 to 6.8% increase in egg production compared to the warmer 2010s (S1-5).

**Figure 8:**
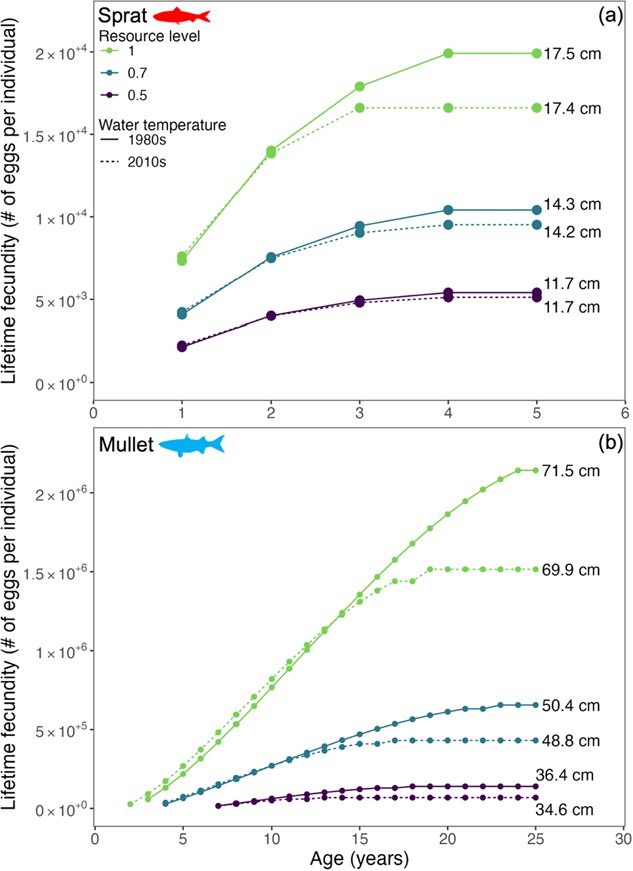
Predicted lifetime fecundity (points) of European sprat (panel a) and thinlip mullet (panel b) under contrasting environmental conditions. Points indicate discrete spawning events and labels display predicted total length. Please note the difference in scaling on the x and y axes. The time axis for sprat is restricted to 5 years because reproduction ceased after age 4.

Sprat was predicted to have lower lifetime fecundity in the 2010s compared to the 1980s (Fig. 8a). Unlike the length of sprat predicted by the model, which was largely controlled by resource level (Fig. 3c), both resource level and temperature regime influenced sprat lifetime fecundity. During the first reproductive year (Year 1), sprat fecundity in the warmer (2010s) temperature was 4% to 5% higher than in the colder (1980s) temperature and only marginally different (1%) in the following year (Year 2). In the first 2 years, egg production was almost exclusively controlled by resource level. Later in life (i.e. years 3–4), resource level still influenced sprat egg production; however, depending on the resource level, production was reduced by 5% to 17% in the 2010s. At the highest (1) resource level, reproduction ceased 2 years earlier, ending after Year 3 in the warmer 2010s.

Temperature and resource level interacted with body size to influence the lifetime fecundity of mullet (Fig. 8b). At the high resource level (1.0), mullet reproduced one year earlier (year two) and produced 58% more eggs under warmer (2010s) versus cooler (1980s) temperatures in year three. Egg production then declined by ~ 5% each subsequent year until a transition period (Year 14), after which fecundity became higher under cooler conditions, peaking 29% above the warmer scenario by Year 25; reproduction ceased under warmer conditions in Year 19. A similar pattern emerged at the intermediate (0.7) resource level (Fig. 8b): initial advantages under warmer conditions (up to 25% higher fecundity through Year 10) reversed after the transition period, with cooler simulations maintaining reproduction longer and reaching 34% higher output by Year 25. At low (0.5) resource levels, fecundity was consistently greater under cooler temperatures, with 51% higher production in the final reproductive year (Year 19), whereas reproduction ended six years earlier (Year 13) under warmer conditions.

## Discussion

Coastal zones serve as critical nursery habitats, providing abundant resources essential for the rapid growth of fish (Beck *et al.*, 2001). However, these shallow-water nurseries face considerable threats from human activities and climate change (Harley *et al.*, 2006; Lotze *et al.*, 2006; Reimann *et al.*, 2023). Understanding the complex interplay between resource level (prey availability) and temperature on fish performance is crucial, as it influences population dynamics and can ultimately reshape community structures (Dahlke *et al.*, 2020; Peck *et al.*, 2021). Our findings on species-specific responses—differing between biogeographical guilds—emphasize the importance of integrating size-dependent thermal performance and resource availability to fully grasp the response of fish species to changing environmental conditions.

### Threats of warming to species at lower latitudinal limits of their distribution

Our model highlights physiological mechanisms limiting the persistence of fishes experiencing warming of habitats at the lower latitudinal limit of the distribution of the species. As an example, cod exists at its lower latitudinal limit in the Wadden Sea and southern North Sea region. Empirical studies by Björnsson and Steinarsson (2002) and Björnsson *et al.* (2007) demonstrated how optimal growth temperatures decrease from 15 to 11°C as cod grow from 6 to 28 cm TL, while high mortality occurs at ≥20°C. Comparing these size-specific temperature optima and constraints with observed warming trends in the western Wadden Sea reveals that the duration of thermally optimal conditions (temperatures ≤11°C) has decreased from 92 days in the 1980s to 66 days in the 2010s (Fig. 2). Moreover, peak summer temperatures in the 2010s approach or exceed the lethal threshold (20°C) for even moderately sized juvenile cod (~16 cm; Björnsson *et al.*, 2007).

Based on declines in abundance of juvenile fish, the nursery function of the Wadden Sea has continually declined since the 1980s; this holds in general, and in particular for marine species, such as cod (Tulp *et al.*, 2022; Tulp *et al.*, 2017; Table 1). This nursery function has even been suggested to have been completely lost (Heessen *et al.*, 2015). Water temperature has always been considered to be a driving mechanism behind the declining use of the Wadden Sea by cod. Since habitat utilization of deeper/cooler areas outside the Wadden Sea has remained stable since the 1990s (Tulp *et al.*, 2017), it appears that cod has relocated offshore to deeper waters with cooler temperatures (Dulvy *et al.*, 2008). An offshore shift in response to warming water temperature has been documented in juveniles of other boreal species such as plaice (*Pleuronectes platessa*, Pleuronectidae; Perry *et al.*, 2005; van Keeken *et al.*, 2007), which historically utilized the Wadden Sea as a nursery. Unfavourable temperatures along the Norwegian Skagerrak coast were postulated to cause cod to select habitats in cooler, deeper waters at the expense of available resources (Freitas *et al.*, 2016). Cod exhibit an ontogenetic diet shift, typically observed at lengths exceeding 30 cm, co-occurring with a habitat shift to greater depths (Link *et al.*, 2009). In the Wadden Sea, juvenile cod rely heavily on brown shrimp (*Crangon crangon*) and a premature shift to deeper, cooler water may expose cod to less suitable or less energy-rich prey. This highlights the importance of temperature but also size-specific resource availability. It is important to note that impacts of fisheries (Cook *et al.*, 1997) may also have contributed to the decline of cod within the Wadden Sea (Tulp *et al.*, 2022). Nevertheless, our simulations predicted that 1+ cod (>10 cm) cannot survive the peak summer temperatures in the 2010s (Figs 2, 3a and 5a), offering mechanistic evidence that warming has transformed the Wadden Sea into an unfavourable nursery area for this species.

### Habitat suitability of coastal migrants

Some fishes perform short-term or seasonal feeding migrations into shallow coastal waters (Gillanders *et al.*, 2003; Able, 2005), and our model sheds light on how this life history strategy can be compromised by climate-driven warming. As an example, herring use the Wadden Sea as marine juveniles, marine adventitious migrants and seasonal migrants (Tulp *et al.*, 2022; Table 1). Based on the abundance in field surveys, the sizes of herring schools using the Wadden Sea increased throughout the 1980s, then stabilized before decreasing since 2005 (Tulp *et al.*, 2022). Maathuis *et al.* (2023) found that the migration pathway between the North Sea (spawning area) and Wadden Sea (nursery area) was largely dominated by small-sized (mean 6.4 cm) herring from March through December, though the possibility that larger herring utilize this habitat during the colder winter months remains speculative due to the absence of winter field sampling. Observations (Maathuis *et al.*, 2023; Rademaker *et al.*, 2024; Fig. 7a) and our model predictions (Fig. 3b) indicate that the western Wadden Sea has become less suitable for larger-sized herring (e.g. marine adventitious and seasonal migrants). Although herring and other fishes migrating to coastal areas are capable of relocating to more suitable water temperatures if those exist and can be found (such as deeper waters), this may not be sufficient to prevent thermal stress. Our model predictions revealed the potential underlying mechanisms. At the maximum resource level, the warmer water temperatures of the 2010s supported fast growth, ultimately causing herring to ‘grow to death’ under these conditions. During the first year, herring length (Fig. 3b) and water temperatures simultaneously increased during the growing season (Fig. 2). As herring grow, their thermal boundaries continually shift towards lower temperatures (S1-3) and this ultimately resulted in starvation mortality as thermal boundaries were reached and fish of larger body size did not have sufficient energy reserves to cover increased metabolic costs incurred during warmer months (Figs 2 and 5b). At moderate and low resource levels within the 2010s simulations, herring persisted into their second year due to a match between their length-specific thermal tolerance and temperature during the peak growing season in Year 1 but not in Year 2 when summer temperatures surpassed tolerance limits.

### Changes in habitat suitability for coastal resident species

Similar to changes in seasonal migrants, changes in the performance of resident species in coastal systems may provide evidence for changes in the habitat suitability. Rockling is a year-round resident of the Wadden Sea, which, unlike many other species in this system, has increased in abundance since the 1990s (Tulp *et al.*, 2022). This increase coincided with the recovery of subtidal beds of blue mussel (*Mytilus edulis*) and Pacific oyster (*Crassostrea gigas*), which likely provided the physical structure rockling prefers in both laboratory (Dye *et al.*, 2024) and field settings (Mulder *et al.*, 2020; Dickson *et al.*, 2023; Watson *et al.*, 2025). Simulations and field data (Figs 4a and 7b; Table 2) both suggest, however, a decrease in length-at-age of rockling with increasing temperature. Reduced length-at-age is unlikely due to resource limitation because the Wadden Sea supports a high biomass of brown shrimp (*C. crangon*), a main prey of rockling. Although fishing pressure on shrimp has increased (Respondek *et al.*, 2022), it is unclear whether brown shrimps in the preferred size classes of rockling have become less abundant (Tulp *et al.*, 2016).

Simulations provide a glimpse into the plausible future body condition of rockling under further increases in seawater temperatures as projected by Kristiansen *et al.* (2024) for the greater North Sea basin and NE Atlantic. Our model predicted reductions in growth, lifespan and lifetime fecundity between the 1980s and 2010s. Continued climate-driven warming will likely lead to further decrements in performance as rockling’s thermal tolerance limits are projected to be further exceeded during the summer months of growing season. Notably, within the warmer (2010s) simulations, rockling no longer attained their typical maximum lifespan of 3–4 years (Cohen *et al.*, 1990; Śmietana, 1992) observed in the cooler (1980s) simulations, but remained under 3 years of age (Fig. 4a). Otolith microchemical analysis could provide insights into the possible existence of growth cessation and reduced lifespan within the Wadden Sea rockling population (Fukuda *et al.*, 2009; Pecquerie *et al.*, 2012; Vaughan *et al.*, 2021).

### Temperature-dependent changes in fecundity with age/size

Although local warming may favour the early growth or survival of species living at or near the higher latitudinal limit of the distribution of species, changes in reproductive potential can occur due to size-specific changes in thermal tolerance. As an example, mullet and sprat were predicted in model runs to have no substantial change in growth but to experience reduced lifetime fecundity in the warmer 2010s compared to the colder 1980s. This reduction in reproductive output with age was attributed to size-dependent increases in maintenance costs at higher temperatures, which diminished the energy available for egg production.

The ability of fishes to allocate sufficient energy resources to fuel reproduction is critical to population persistence (Rijnsdorp *et al.*, 2010). In the North Sea, annual sprat fecundity ranges from ~1000 to 4000 eggs per gram of female (Bailey and Pipe, 1977; Alheit, 1987). These empirical observations align with our model predictions, where simulated sprat weights (6 to 19 g) correspond to fecundity estimates ranging from 6000 to 19 000 eggs per individual (Fig. 8a). Our finding that lifetime fecundity plateaus at increasing water temperature is further supported by empirical evidence from Baltic sprat (Haslob *et al.*, 2011). In addition, it is possible that temperature-related impacts on sprat early life stages have already occurred given that the 2010s’ mid to peak summer temperatures (Fig. 2) exceed sprat early life stages’ thermal maximum (~17°C) for survival (Peck *et al.*, 2012).

### Temperature-size rule

The temperature-size rule (TSR; Atkinson, 1994) describes the response of ectothermic organisms to increasing environmental temperature, resulting in faster growth and development but reduced adult body size. Our model predictions do not unequivocally support the TSR. Instead, the model predictions highlight that observations corroborating the TSR are contingent on the relation between the species-specific (and size-specific) thermal requirements and the degree of warming in relation to the previously experienced (ambient) environment (Ohlberger, 2013; Huss *et al.*, 2019). Consistent with the TSR, our model predicted reductions in maximum length of rockling and mullet during the warmer 2010s. However, these reductions did not occur in all of the species simulated, nor were they guild specific as mullet and rockling belong to the Lusitanian and boreal guilds, respectively (Fig. 4 and Table 1). Thermal safety margins, characterized by the temperature difference between an optimum and environmental temperature, can shield species from the negative impacts of temperature increases (Ohlberger, 2013). Sprat experience this thermal safety margin as model predictions show comparable growth for the 1980s and 2010s (Fig. 3c). However, this thermal safety margin does not encompass the reproductive output of sprat (Fig. 8a). In the Baltic Sea, juvenile sprat seldom attain their optimal growth temperatures (Peck *et al.*, 2012), suggesting a thermal safety margin exists within this species whose biomass and recruitment have benefited from increasing water temperature (Peck *et al.*, 2013; Peck *et al.*, 2021). Irrespective of the model species, increasing environmental temperature did not generally increase predicted growth rates during early life as suggested by the TSR (Daufresne *et al.*, 2009; Baudron *et al.*, 2014; Lindmark *et al.*, 2022). Support for the TSR was only evident in mullet, which reached maturity faster for a higher seasonal temperature, thereby reproducing 1 year earlier in simulations with 2010s temperature (Fig. 8b). Further increases in environmental temperatures will exacerbate existing negative impacts and may give rise to novel detrimental effects (Lindmark *et al.*, 2018), particularly within the Lusitanian species that are increasing in the northern reaches of their distributional range.

### Model framework

Our model is not based on the conventional assumption that physiological rates uniformly respond to changes in temperature (Kooijman, 2010; but see Frisk *et al.*, 2015) but accounts for species-specific and size-dependent optimal and maximum temperatures for rates of ingestion and maintenance metabolism (as detailed in Table 3 and S1, 3-4). This tailored methodology yields a more nuanced (i.e. size-specific) understanding of how temperature influences physiological functions, thereby enhancing the accuracy and reliability of the model predictions, particularly in situations where thermal conditions are suboptimal for specific sizes. This is a step forward but highlights a current challenge in temperature-dependent modelling applications. For many species, in particular species of low commercial interest, empirical data (e.g. growth, temperature tolerances) are simply scarce (Freitas *et al.*, 2010). If data are available, it is generally restricted to specific life stages (Catalán *et al.*, 2019). Future research should aim to better understand individual-level physiological responses by conducting species-specific growth studies. It is also essential to evaluate how fish cope with multiple environmental stressors and explore critical factors such as energy allocation strategies in reproducing adults (McBride *et al.*, 2015; Cominassi *et al.*, 2020).

The model is grounded in first principles, and the parameters reflect known physiological traits rather than generalized assumptions. The simulations were used as testable predictions on individual performance that we corroborated both qualitatively and quantitatively using species-specific findings from field surveys and monitoring programmes (see Table 2). The general *post hoc* agreement among the major outcomes and empirical findings indicates that the mechanistic processes and parameter estimates reliably represent the model species. Some discrepancies existed between simulated and observed length-at-age data, which is not unexpected. For example, the use of Wadden Sea seasonal temperature inputs may have contributed to higher predicted growth rates at smaller sizes for herring and sprat, as these were compared to observations from the colder central North Sea. Although fishing can affect population dynamics similarly to warming-driven change (Gårdmark and Huss, 2020), there is little evidence for such effects in our study species except for cod where our results reflect temperature-driven processes. The model is deterministic (nonstochastic) and generates output for three fixed levels of resource availability, whereas field data on length-at-age lack direct information about the (resource) environment experienced by individual fish.

Accurately characterizing resource availability to fish (and other organisms in the field) remains a challenge. While some biogenetics-based approaches (Brownscombe *et al.*, 2022) estimate fish resource consumption from field-derived growth rates, this approach was not taken here. Unlike temperature—which is relatively easy to measure and model—resource availability is highly variable across space and time, and is often subject to methodological limitations (Ouellet *et al.*, 2025). Common proxies such as chlorophyll-a concentration may not reliably represent the biomass available to higher trophic levels (Philippart *et al.*, 2007). Moreover, such measures may fail to capture the resource base relevant to fish across different life stages, as many species undergo ontogenetic dietary shifts (Sánchez-Hernández *et al.*, 2019).

Uncertainty in resource levels can influence species performance predictions, as higher or lower assumed resource availability may lead to over- or underestimation of growth and thermal tolerance. Rather than fitting the model to observations with unknown environmental attributes (e.g. resource level), we used simulations at low (0.5) and high (1.0) resource levels to represent the lower and upper bounds of potential environmental conditions. The strong agreement between these simulated trajectories and the observed range in length-at-age (Figs 3 and 4) supports the model’s robustness and its ability to capture the variability imposed by contrasting environmental conditions. Field observations of individuals of the same age can still offer valuable insights. Smaller individuals likely experienced suboptimal conditions, while larger ones may have grown under more favourable circumstances (Persson *et al.*, 2007). These outcomes reflect the influence of various interacting factors, including temperature, resource availability, maternal effects and density-dependent processes (Haugen *et al.*, 2007)—many of which are difficult to quantify in the field and vary across space and time (Ouellet *et al.*, 2025). While incorporating potential thermal adaptation into models is possible, the lack of empirical data, apart from well-studied species like cod (Skjærven *et al.*, 2024), currently limits the feasibility of implementing such mechanisms in our framework. Advancing our understanding of resource dynamics and prey interactions is therefore essential for accurate predictions of fish performance under climate change.

The model presented here provides a general approach that can be broadly applied to other species, geographic areas and research questions. A key strength of this model is its ability to illustrate how seasonal temperature regimes, including changes in both the mean and seasonal amplitude of water temperature, shape fish performance. It provides a platform to explore the effects of future (projected) temperatures (Moreira *et al.*, 2022) and extreme events such as marine heatwaves (Wethey and Woodin, 2022) on species- and size-specific performance. While we used observed differences in seasonal temperature regimes between the 1980s and 2010s as an indication of projected climate change impacts, our results indicate that variation in resource availability had a greater influence on predicted vital rates than temperature alone. This highlights the critical need to better quantify resource levels in order to improve predictions of organismal performance and responses to environmental change (Railsback, 2022).

To improve species-specific insights and projections of the direct and indirect effects of climate warming, this modelling framework can be readily integrated to account for population-level processes and feedbacks (van de Wolfshaar *et al.*, 2008; Ohlberger *et al.*, 2011), as well as trophic interactions (van Leeuwen *et al.*, 2014; Soudijn and de Roos, 2017). A simple example is the model by Akimova *et al.* (2016) examining the growth and survival of cod larvae and juveniles based on the spatiotemporal overlap with predators in the North Sea where temperature-driven increases in somatic growth rate were less than those for the consumption rates of predators, causing faster growth but lower survival. Incorporating this type of spatiotemporal dynamics with predators or potential prey items via ecosystem model outputs (Teal *et al.*, 2012) or applying mechanistic species distribution modelling (Kearney and Porter, 2009; Rose *et al.*, 2024) is an important next step. Such integration could help identify size- and habitat-specific vulnerabilities to warming, thereby informing management decisions by highlighting the mechanisms behind a population’s sensitivity to environmental change. This will allow a better understanding of size-specific temperature responses (Peck *et al.*, 2021) and help identify how environmental change creates bottlenecks to the performance and life cycle closure of fish (Petitgas *et al.*, 2013) and other organisms.

Overall, our findings highlight that while resource availability remains the dominant driver of fish performance, temperature imposes important physiological constraints, particularly when species approach their thermal limits. The contrasting outcomes among species demonstrate that warming will not affect all species equally, and that even modest increases in water temperature can alter growth, energy allocation and key life-history processes.

## Supplementary Material

Web_Material_coag005

## Data Availability

The data and code that support the findings of this study are available at https://github.com/bassdye/Dye_et_al_ind_level_temp_model.
