# Correction to: Disentangling the effects of resource level and temperature dependence on the performance of fish in different guilds

**DOI:** 10.1093/conphys/coag050

**Published:** 2026-07-22

This is a correction to: Bass Dye, Myron A Peck, Karen E van de Wolfshaar, Anieke van Leeuwen, Disentangling the effects of resource level and temperature dependence on the performance of fish in different guilds, *Conservation Physiology*, Volume 14, Issue 1, 2026, coag005, 10.1093/conphys/coag005.

In the originally published version of this manuscript, the letter labelling in Table 1 was incorrectly ordered.

Table 1 has been corrected to read:

**Table 1:** Species parameterized in the generic physiological model including their guilds and characteristic life-history stage in the Wadden Sea

Species	Common name	Biogeographic guild	Functional guild	Wadden Sea life stage
*Gadus morhua*	Atlantic cod	Boreal^a^	Marine juvenile^c^	Juvenile
*Clupea harengus*	Atlantic herring	Boreal	Marine juvenile Marine seasonal migrant^d^ Marine adventitious^e^	Juvenile - adult
*Ciliata mustela*	Five bearded rockling	Boreal	Estuarine resident^f^	Juvenile - adult
*Sprattus sprattus*	European sprat	Lusitanian^b^	Marine seasonal migrant Marine adventitious	Juvenile - adult
*Chelon ramada*	Thinlip mullet	Lusitanian	Opportunist^g^	Juvenile - adult

Biogeographic guilds follow Jiming (1982), while functional guilds are defined according to Elliott and Hemingway (2002) and Elliott et al. (2007).

^a^Distribution centered north of the English Channel (cold-water affinity).

^b^Distribution centered south of the English Channel (warm-water affinity).

^c^Regularly enter estuaries but use nearshore marine waters as alternative habitat.

^d^Enter estuaries in large numbers as juveniles.

^e^Enter estuaries during certain times of the year.

^f^Occasionally (irregularly) enter estuaries, most frequently occur in high salinity (~35 PSU), lower reaches and coastal marine waters.

^g^Complete (or capable of) entire life cycle within an estuarine environment.

Instead of:

**Table 1:** Species parameterized in the generic physiological model including their guilds and characteristic life-history stage in the Wadden Sea

Species	Common name	Biogeographic guild	Functional guild	Wadden Sea life stage
*G. morhua*	Atlantic cod	Boreal^a^	Marine juvenile^c^	Juvenile
*C. harengus*	Atlantic herring	Boreal	Marine juvenile Marine seasonal migrant^d^ Marine adventitious^e^	Juvenile–adult
*C. mustela*	Five bearded rockling	Boreal	Estuarine resident^f^	Juvenile–adult
*S. sprattus*	European sprat	Lusitanian^b^	Marine seasonal migrant Marine adventitious	Juvenile–adult
*C. ramada*	Thinlip mullet	Lusitanian	Opportunist^g^	Juvenile–adult

Biogeographic guilds follow Jiming (1982), while functional guilds are defined according to Elliott and Hemingway (2002) and Elliott *et al.* (2007).

^a^Distribution centred north of the English Channel (cold-water affinity).

^b^Regularly enter estuaries but use nearshore marine waters as alternative habitat.

^c^Enter estuaries in large numbers as juveniles.

^d^Enter estuaries during certain times of the year.

^e^Occasionally (irregularly) enter estuaries, most frequently occur in high salinity (~35 PSU), lower reaches and coastal marine waters.

^f^Complete (or capable of) entire life cycle within an estuarine environment.

^g^Distribution centred south of the English Channel (warm-water affinity).

Additionally, equations in Table 4 have been emended and corrected to read:

**Table 4:** Individual-level model variables and equations

Subject	Equation
Standardized mass (g)	$\mathrm{m}\left(\mathrm{x}\right)=\mathrm{x}\left(1+{\mathrm{q}}_{\mathrm{j}}\right)$
Total mass (g)	$\mathrm{w}\left(\mathrm{x},\mathrm{y}\right)=\mathrm{x}+\mathrm{y}$
Body length (cm)	$\mathrm{L}\left(\mathrm{x}\right)={\lambda}_1\mathrm{m}{\left(\mathrm{x}\right)}^{\lambda_2}$
Intake (g day^-1^)	$\mathrm{I}\left(\mathrm{x},\mathrm{T}\right)={\mathrm{d}}_1\mathrm{m}{\left(\mathrm{x}\right)}^{{\mathrm{d}}_2}{\mathrm{r}}_{\mathrm{a}}\left(\mathrm{x},\mathrm{T}\right){\mathrm{R}}_{\mathrm{l}}$
Juvenile: energy allocation to x when L < L_m_	$\mathrm{f}\left(\mathrm{x},\mathrm{y}\right)=\left\{\begin{array}{c}\frac{1}{\left(1+{\mathrm{q}}_{\mathrm{j}}\right){\mathrm{q}}_{\mathrm{j}}^2}{\left(\frac{\mathrm{y}}{\mathrm{x}}\right)}^2\kern0.5em \mathrm{if}\ \frac{\mathrm{y}}{\mathrm{x}}<{\mathrm{q}}_{\mathrm{j}}\\{}\frac{1}{1+{\mathrm{q}}_{\mathrm{j}}}\kern3.75em \mathrm{otherwise}\end{array}\right.$
Adult: energy allocation to x when L > L_m_	$\mathrm{f}\left(\mathrm{x},\mathrm{y}\right)=\left\{\begin{array}{c}\frac{1}{\left(1+{\mathrm{q}}_{\mathrm{a}}\right){\mathrm{q}}_{\mathrm{a}}^2}{\left(\frac{\mathrm{y}}{\mathrm{x}}\right)}^2\kern0.5em \mathrm{if}\ \frac{\mathrm{y}}{\mathrm{x}}<{\mathrm{q}}_{\mathrm{a}}\\{}\frac{1}{1+{\mathrm{q}}_{\mathrm{a}}}\kern3.75em \mathrm{otherwise}\end{array}\right.$
Acquired energy (g day^-1^)	${\mathrm{E}}_{\mathrm{a}}\left(\mathrm{x},\mathrm{T}\right)={\mathrm{k}}_{\mathrm{e}}\mathrm{I}\left(\mathrm{x},\mathrm{T}\right)$
Energy requirements for maintenance (g day^-1^)	${\mathrm{E}}_{\mathrm{m}}\left(\mathrm{x},\mathrm{T}\right)={\rho}_1{\left(\mathrm{m}\left(\mathrm{x}\right)\right)}^{\rho_2}{\mathrm{r}}_{\mathrm{m}}\left(\mathrm{x},\mathrm{T}\right)$
Energy balance (g day^-1^)	${\mathrm{E}}_{\mathrm{g}}\left(\mathrm{x},\mathrm{T}\right)={\mathrm{E}}_{\mathrm{a}}\left(\mathrm{x},\mathrm{T}\right)-{\mathrm{E}}_{\mathrm{m}}\left(\mathrm{x},\mathrm{T}\right)$
Fecundity (#)	$\mathrm{F}\left(\mathrm{x},\mathrm{y}\right)=\left\{\begin{array}{c}\frac{{\mathrm{k}}_{\mathrm{r}}\left(\mathrm{y}-{\mathrm{q}}_{\mathrm{j}}\mathrm{x}\right)}{w_{\mathrm{b}}}\kern0.5em \mathrm{if}\ \mathrm{L}>{\mathrm{L}}_{\mathrm{m}}\ \mathrm{and}\ \mathrm{y}>{\mathrm{q}}_{\mathrm{j}}\mathrm{x}\\{}0\kern0.5em \mathrm{otherwise}\end{array}\right.$
Temperature dependence	$\mathrm{r}\left(\mathrm{x},\mathrm{T}\right)=\mathrm{V}{\left(\mathrm{x},\mathrm{T}\right)}^{\mathrm{X}\left(\mathrm{x},\, \mathrm{T}\right)}{\mathrm{e}}^{\mathrm{X}\left(\mathrm{x},\, \mathrm{T}\right)\left[1-\mathrm{V}\left(\mathrm{x},\,\mathrm{T}\right)\right]}$
	$\mathrm{V}\left(\mathrm{x},\mathrm{T}\right)=\frac{\left({\mathrm{T}}_{\mathrm{max}}\left(\mathrm{x}\right)-\mathrm{T}\right)}{\left({\mathrm{T}}_{\mathrm{max}}\left(\mathrm{x}\right)-{\mathrm{T}}_{\mathrm{opt}}\left(\mathrm{x}\right)\right)}$
	$\mathrm{X}\left(\mathrm{x},\mathrm{T}\right)={\mathrm{W}}^2{\left[1+{\left(1+\frac{40}{\mathrm{Y}}\right)}^{0.5}\right]}^2\frac{1}{400}$
	$\mathrm{W}=\left({\mathrm{T}}_{\mathrm{max}}\left(\mathrm{x}\right)-{\mathrm{T}}_{\mathrm{opt}}\left(\mathrm{x}\right)\right)\ln\!\mathrm{Q}(\mathrm{x})$
	$\mathrm{Y}=\left({\mathrm{T}}_{\mathrm{max}}\left(\mathrm{x}\right)-{\mathrm{T}}_{\mathrm{opt}}\left(\mathrm{x}\right)+2\right) \ln\!\mathrm{Q}(\mathrm{x})$
	${\mathrm{T}}_{\mathrm{opt}}\left(\mathrm{x}\right)={\gamma}_{\mathrm{opt}}{\left(\mathrm{m}\left(\mathrm{x}\right)\right)}^{{\mathrm{v}}_{\mathrm{opt}}}$
	${\mathrm{T}}_{\mathrm{max}}\left(\mathrm{x}\right)={\gamma}_{\mathrm{max}}{\left(\mathrm{m}\left(\mathrm{x}\right)\right)}^{{\mathrm{v}}_{\mathrm{max}}}$
	$\mathrm{Q}\left(\mathrm{x}\right)=\vartheta{\left(\mathrm{m}\left(\mathrm{x}\right)\right)}^{\theta}$
Acquired energy (°C)	${\mathrm{T}}_{\mathrm{a},\, \mathrm{opt}}\left(\mathrm{x}\right)={\gamma}_{\mathrm{a},\mathrm{opt}}{\left(\mathrm{m}\left(\mathrm{x}\right)\right)}^{{\mathrm{v}}_{\mathrm{a},\mathrm{opt}}}$
	${\mathrm{T}}_{\mathrm{a},\, \max}\left(\mathrm{x}\right)={\gamma}_{\mathrm{a},\max }{\left(\mathrm{m}\left(\mathrm{x}\right)\right)}^{{\mathrm{v}}_{\mathrm{a},\max }}$
	${\mathrm{Q}}_{\mathrm{a}}\left(\mathrm{x}\right)={\vartheta}_{\mathrm{a}}{\left(\mathrm{m}\left(\mathrm{x}\right)\right)}^{\theta_{\mathrm{a}}}$
Metabolism (°C)	${\mathrm{T}}_{\mathrm{m},\mathrm{opt}}\left(\mathrm{x}\right)={\gamma}_{\mathrm{m},\mathrm{opt}}{\left(\mathrm{m}\left(\mathrm{x}\right)\right)}^{{\mathrm{v}}_{\mathrm{m},\mathrm{opt}}}$
	${\mathrm{T}}_{\mathrm{m},\max}\left(\mathrm{x}\right)={\gamma}_{\mathrm{m},\max }{\left(\mathrm{m}\left(\mathrm{x}\right)\right)}^{{\mathrm{v}}_{\mathrm{m},\max }}$
	${\mathrm{Q}}_{\mathrm{m}}\left(\mathrm{x}\right)={\vartheta}_{\mathrm{m}}{\left(\mathrm{m}\left(\mathrm{x}\right)\right)}^{\theta_{\mathrm{m}}}$
State variable dynamics	
Growth in structural mass, x	$\frac{\mathrm{dx}}{\mathrm{dt}}=\left\{\begin{array}{c}\mathrm{f}\left(\mathrm{x},\mathrm{y}\right){\mathrm{E}}_{\mathrm{g}}\left(\mathrm{x},\mathrm{T}\right)\kern4.00em \mathrm{if}\ {\mathrm{E}}_{\mathrm{g}}>0\\{}0\kern9.25em \mathrm{otherwise}\end{array}\right.$
Growth in reserve mass, y	$\frac{\mathrm{dy}}{\mathrm{dt}}=\left\{\begin{array}{c}\left(1-\mathrm{f}\left(\mathrm{x},\mathrm{y}\right)\right){\mathrm{E}}_{\mathrm{g}}\left(\mathrm{x},\mathrm{T}\right)\kern3.00em \mathrm{if}\ {\mathrm{E}}_{\mathrm{g}}>0\\{}{\mathrm{E}}_{\mathrm{g}}\left(\mathrm{x},\mathrm{T}\right)\kern8em \mathrm{otherwise}\end{array}\right.$

Instead of:

**Table 4:** Individual-level model variables and equations

Subject	Equations
Standardized mass (g)	$m(x)=x\left(1+{q}_j\right)$
Total mass (g)	$w(x)=x+y$
Body length (cm)	$L(x)={\lambda}_1m{(x)}^{\lambda_2}$
Intake (g day^−1^)	$I\left(x,y,T\right)={d}_1m{(x)}^{d_2}{r}_a\left(m(x),T\right){R}_l$
Juvenile: energy allocation to *x* when *L* < *L*_m_	$f\left(x,y\right)=\left\{\begin{array}{c}\frac{1}{\left(1+{q}_j\right){q}_j^2}{\left(\frac{y}{x}\right)}^2\kern0.5em if\ \frac{y}{x}<{q}_j\\{}\frac{1}{1+{q}_j}\kern3.75em \mathrm{otherwise}\end{array}\right.$
Adult: energy allocation to *x* when *L* > *L*_m_	$f\left(x,y\right)=\left\{\begin{array}{c}\frac{1}{\left(1+{q}_a\right){q}_a^2}{\left(\frac{y}{x}\right)}^2\kern0.5em if\ \frac{y}{x}<{q}_a\\{}\frac{1}{1+{q}_a}\kern3.75em \mathrm{otherwise}\end{array}\right.$
Acquired energy (g day^−1^)	${E}_a\left(x,y,T\right)={k}_eI\left(x,y,T\right)$
Energy requirements for maintenance (g day^−1^)	${E}_m\left(x,y,T\right)={\rho}_1{\left(x+y\right)}^{\rho_2}{r}_m\left(m(x),T\right)$
Energy balance (g day^−1^)	${E}_g\left(x,y,T\right)={E}_a\left(x,y,T\right)-{E}_m\left(x,y,T\right)$
Fecundity (#)	$F\left(x,y\right)=\left\{\begin{array}{ll}\frac{k_r\left(y-{q}_jx\right)}{w_b}\kern0.5em if\ L>{L}_m\ \mathrm{and}\ y>{q}_jx\\{}0\kern3.75em \mathrm{otherwise}\end{array}\right.$
Temperature dependence	$r\left(x,y,T\right)=V{\left(x,y,T\right)}^{X\left(x,y,T\right)}{e}^{X\left(x,y,T\right)\left[1-V\left(x,y,T\right)\right]}$
	$V\left(x,y,T\right)=\frac{\left({T}_{\mathrm{max}}\left(x,y\right)-T\right)}{\left({T}_{\mathrm{max}}\left(x,y\right)-{T}_{\mathrm{opt}}\left(x,y\right)\right)}$
	$X\left(x,y,T\right)={W}^2{\left[1+{\left(1+\frac{40}{Y}\right)}^{0.5}\right]}^2\frac{1}{400}$
	$W=\left({T}_{\mathrm{max}}\left(x,y\right)-{T}_{\mathrm{opt}}\left(x,y\right)\right)\ \mathit{\ln}\ Q\left(x,y\right)$
	$Y=\left({T}_{\mathrm{max}}\left(x,y\right)-{T}_{\mathrm{opt}}\left(x,y\right)+2\right)\ \mathit{\ln}\ Q\left(x,y\right)$
	${T}_{\mathrm{opt}}\left(x,y\right)={\gamma}_{\mathrm{opt}}{\left(x+y\right)}^{v_{\mathrm{opt}}}$
	$T_{max}$ $\left(x,y\right)={\gamma}_{\mathrm{max}}{\left(x+y\right)}^{v_{\mathrm{max}}}$
	$Q\left(x,y\right)=\vartheta{\left(x+y\right)}^{\theta }$
Acquired energy (°C)	$T$ $_{a,\mathrm{opt}}\left(x,y\right)=\gamma_{a,\mathrm{opt}}$ ${\left(x+y\right)}^{v_{a,\mathrm{opt}}}$
	$T_{a,\mathrm{max}}$ $\left(x,y\right)=\gamma_{a,\mathrm{max}}$ $\left(x+y\right) v_{a,\mathrm{max}}$
	${Q}_a\left(x,y\right)={\vartheta}_a{\left(x+y\right)}^{\theta_a}$
Metabolism (°C)	$T_{m,\mathrm{opt}}$ $\left(x,y\right)=\gamma_{m,\mathrm{opt}}$ $\left(x+y\right) v_{m,\mathrm{opt}}$
	$T_{m,\mathrm{max}}\left(x,y\right)=\gamma_{m,\mathrm{max}}{\left(x+y\right)}^{v_{m,\mathrm{max}}}$
	${Q}_m\left(x,y\right)={\vartheta}_m{\left(x+y\right)}^{\theta_m}$
State variable dynamics	
Growth in structural mass, *x*	$\frac{dx}{dt}=\left\{\begin{array}{c}f\left(x,y\right){E}_g\left(x,y,T\right)\kern3.00em if\ {E}_g>0\\{}0\kern9.25em \mathrm{otherwise}\end{array}\right.$
Growth in reserve mass, *y*	$\frac{dy}{dt}=\left\{\begin{array}{c}\left(1-f\left(x,y\right)\right){E}_g\left(x,y,T\right)\kern3.00em if\ {E}_g>0\\{}{E}_g\left(x,y,T\right)\kern8em \mathrm{otherwise}\end{array}\right.$

