# Supplementary material for: Disentangling the effects of resource level and temperature dependence on the performance of fish in different guilds
Source: Conserv Physiol. 2026 Jan 28;14(1):coag005. doi: 10.1093/conphys/coag005 (PMC12851634; doi:10.1093/conphys/coag005)
Supplement: Web_Material_coag005 [file web_material_coag005.pdf]

Supplemental - Dye et al. temperature dependent individual level model

Overview

- The effects of temperature and resource level on individual level processes were investigated using an individual level energy budget model based on (Persson et al. 1998) and temperature dependent parameterization in (Ohlberger et al. 2011).
- This notebook contains the allometric consumption and temperature dependent parameterization procedure for the modelled species:

  Atlantic herring (*Clupea harengus*)
  European sprat (*Sprattus sprattus*)
  Five bearded rockling (*Ciliata mustela*)
  Atlantic cod (*Gadus morhua*)
  Thinlip mullet (*Chelon ramada*)

1. Consumption allometric scaler and exponent parameterization

Overview

- **User-defined fitting protocol for the consumption allometric scaler and exponent:**
  1. **Fit the Maximum Consumption Line**:
     - Use the consumption function along with user-defined allometric scaler and exponent values.
     - Follow the predefined fitting steps 2, 3, and 4.
  2. **Utilize Species-Specific Parameters**:
     - Extract necessary parameters from Table 3 in the main text.
     - Obtain common and maximum lengths for the species from Fishbase.
  3. **Set Maximum Consumption**:
     - **Objective**: Ensure the maximum consumption line is at least 2 times the maximum maintenance level (represented by the yellow line) until the maturation length (indicated by the red dashed line on the plot).
     - After reaching the common length (indicated by the blue dashed line), consumption should start to decrease.
  4. **Plotting Consumption and Maintenance**:
     - Ensure the consumption and maintenance lines intersect at or near the maximum size.

  By following this protocol, you can accurately parameterize the consumption allometric scaler and exponent for the specific species.

Install and load packages

```r
# House keeping; clean environment
rm(list=ls())

# Load packages
if(!require(tidyverse))install.packages("tidyverse")
if(!require(ggplot2))install.packages("ggplot2")
if(!require(viridis))install.packages("viridis")
library("tidyverse")
library("ggplot2")
library("viridis")
```

Functions

```r
# Length & weight relationships
weight_to_length <- function(l1 = weight_length_constant, l2 = weight_length_exp, w){
  l1*w^l2
}

length_to_weight <- function(l1 = weight_length_constant, l2 = weight_length_exp, l){
  (l/l1)^(1/l2)
}

# Maintenance function
maintenance <- function(x) {
  qj <- QJ
  standard_weight <- x * (1 + qj)
  maint_s <- allometric_scalar
  maint_e <- allometric_exp
  temp <- maint_s*standard_weight^maint_e
  return(temp)
}

# Consumption function
consumption <- function(x) {
  qj <- QJ
  standard_weight <- x * (1 + qj)
  conv_eff <- 0.5
  temp <- consumption_scalar*standard_weight^consumption_exp
  return(temp)
}
```

Herring (*Clupea harengus*)

Parameters

```r
species <- "Herring"
weight_length_constant <- 5.65
weight_length_exp <- 0.32
QJ <- 0.7
allometric_scalar <- 0.03
allometric_exp <- 0.8
maturation_length <- 14
common_length <- 30 # From Fish base
max_size <- length_to_weight(l = 45) # From Fish base
com_size <- length_to_weight(l = common_length)
mat_size <- length_to_weight(l = maturation_length)
```

Parameters fitted by visual observation using consumption function

```r
consumption_scalar <- 0.97
consumption_exp <- 0.303
```

Plot

Plot consumption_scalar and consumption_exp parameters to ensure compliance with user-defined fitting protocol.

```r
  ggplot(data = data.frame(x = 0), mapping = aes(x = x)) +
    stat_function(fun = consumption, args = list(), size = 1, lty = "dashed",
                  aes(colour = "Max consumption")) +
    stat_function(fun = maintenance, args = list(), size = 1,
                  aes(colour = "Max maintenance")) +
    geom_vline(xintercept = mat_size, color = "red", linetype = "dashed") +
    geom_vline(xintercept = com_size, color = "blue", linetype = "dashed") +
    theme_bw() +
    labs(subtitle = paste(species), x = "Weight (g)", y = "Rate", color = "") +
    scale_color_viridis(discrete = TRUE) +
    scale_x_continuous(expand = c(0, 0), limits = c(0, max_size))
```

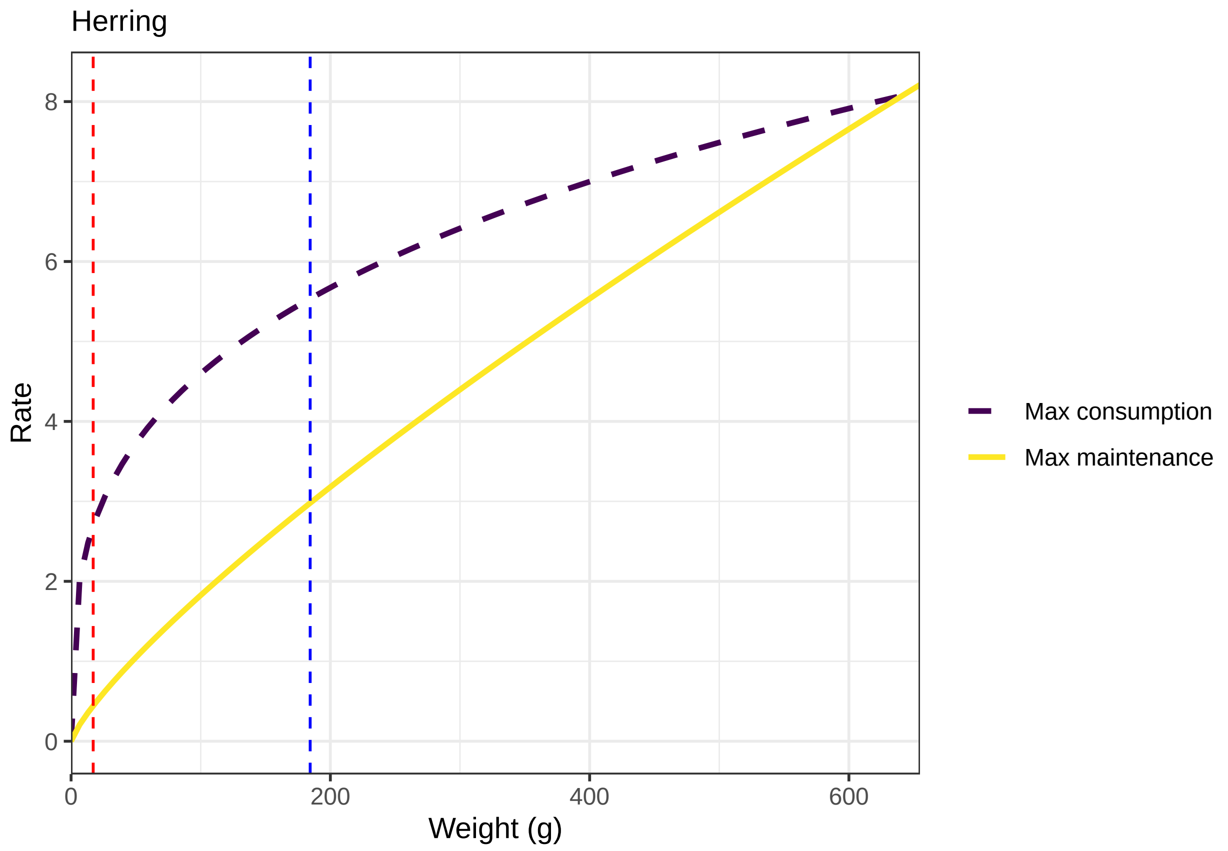

House keeping; clean environment for next species parameterization

```
rm(list = setdiff(ls(), c("consumption", "maintenance", "length_to_weight", "weight_to_length")))
```

Sprat (*Sprattus sprattus*)

Parameters

```
species <- "Sprat"
weight_length_constant <- 5.4
weight_length_exp <- 0.33
QJ <- 0.9
allometric_scalar <- 0.03
allometric_exp <- 0.8
maturation_length <- 9
common_length <- 12  # From Fish base
max_size <- length_to_weight(l = 27) # From Fish base
com_size <- length_to_weight(l = common_length)
mat_size <- length_to_weight(l = maturation_length)
```

Parameters fitted by visual observation using consumption function

```
consumption_scalar <- 0.46
consumption_exp <- 0.215
```

Plot

Plot consumption_scalar and consumption_exp parameters to ensure compliance with user-defined fitting protocol.

```
  ggplot(data = data.frame(x = 0), mapping = aes(x = x)) +
    stat_function(fun = consumption, args = list(), size = 1, lty = "dashed",
                  aes(colour = "Max consumption")) +
    stat_function(fun = maintenance, args = list(), size = 1,
                  aes(colour = "Max maintenance")) +
    geom_vline(xintercept = mat_size, color = "red", linetype = "dashed") +
    geom_vline(xintercept = com_size, color = "blue", linetype = "dashed") +
    theme_bw() +
    labs(subtitle = paste(species), x = "Weight (g)", y = "Rate", color = "") +
    scale_color_viridis(discrete = TRUE) +
    scale_x_continuous(expand = c(0, 0), limits = c(0, max_size))
```

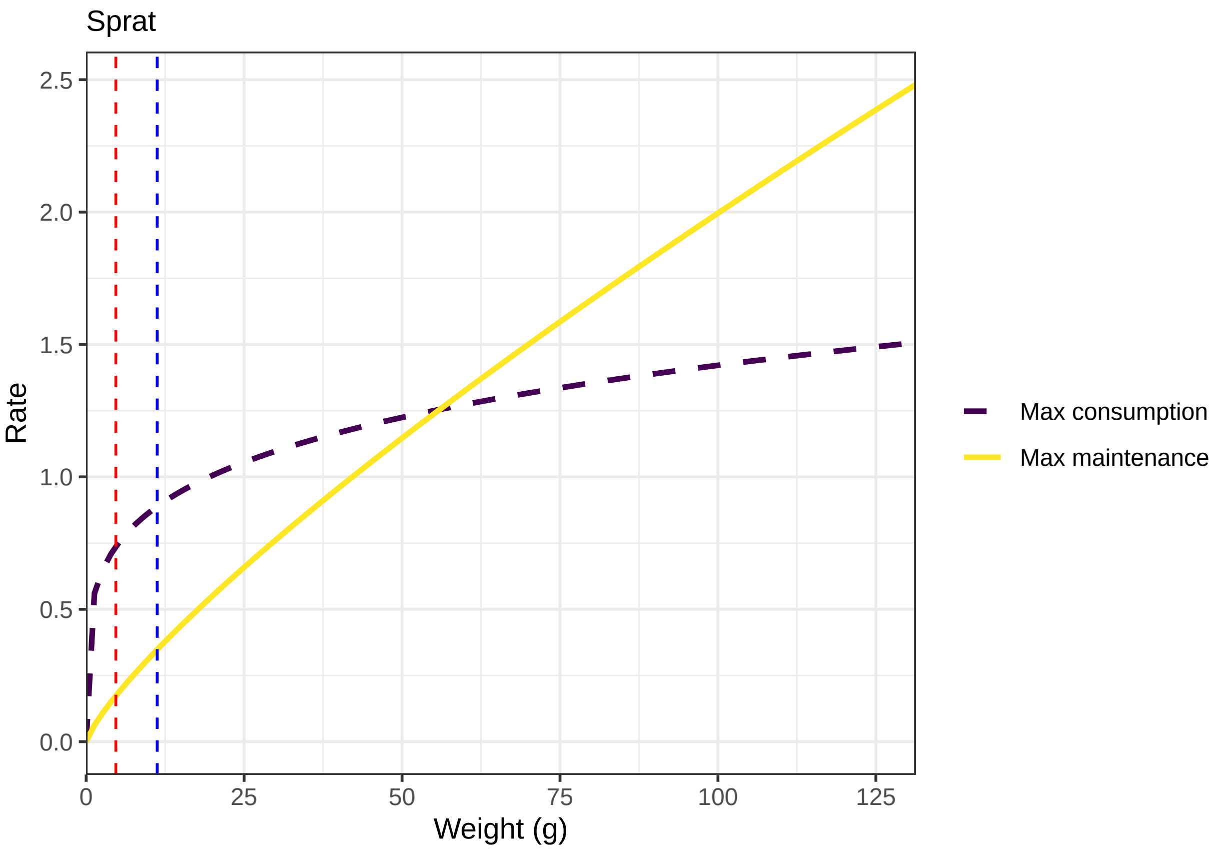

House keeping; clean environment for next species parameterization

```
rm(list = setdiff(ls(), c("consumption", "maintenance", "length_to_weight", "weight_to_length")))
```

Fivebeard rockling (*Ciliata mustela*)

Parameters

```
species <- "Rockling"
weight_length_constant <- 5.26
weight_length_exp <- 0.3174
QJ <- 0.8
allometric_scalar <- 0.03
allometric_exp <- 0.79
maturation_length <- 14
common_length <- 20  # From Fish base
max_size <- length_to_weight(l = 25)  # From Fish base
com_size <- length_to_weight(l = common_length)
mat_size <- length_to_weight(l = maturation_length)
```

Parameters fitted by visual observation using consumption function

```
consumption_scalar <- 1.8
consumption_exp <- 0.05
```

Plot

Plot consumption_scalar and consumption_exp parameters to ensure compliance with user-defined fitting protocol.

```
  ggplot(data = data.frame(x = 0), mapping = aes(x = x)) +
    stat_function(fun = consumption, args = list(), size = 1, lty = "dashed",
                  aes(colour = "Max consumption")) +
    stat_function(fun = maintenance, args = list(), size = 1,
                  aes(colour = "Max maintenance")) +
    geom_vline(xintercept = mat_size, color = "red", linetype = "dashed") +
    geom_vline(xintercept = com_size, color = "blue", linetype = "dashed") +
    theme_bw() +
    labs(subtitle = paste(species), x = "Weight (g)", y = "Rate", color = "") +
    scale_color_viridis(discrete = TRUE) +
    scale_x_continuous(expand = c(0, 0), limits = c(0, max_size))
```

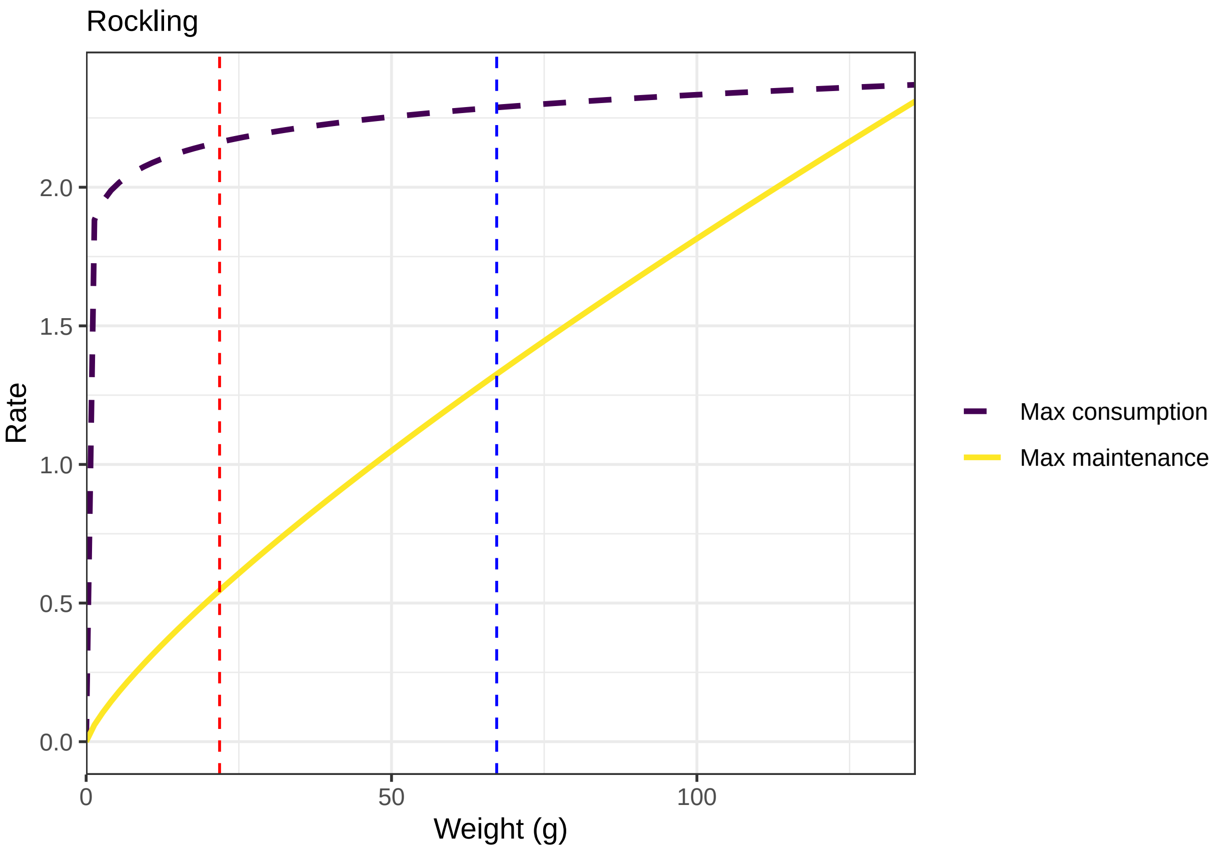

House keeping; clean environment for next species parameterization

```
rm(list = setdiff(ls(), c("consumption", "maintenance", "length_to_weight", "weight_to_length")))
```

Cod (*Gadus morhua*)

Parameters

```
species <- "Cod"
weight_length_constant <- 4.95
weight_length_exp <- 0.325
QJ <- 0.7
allometric_scalar <- 0.03
allometric_exp <- 0.8
maturation_length <- 30
common_length <- 100  # From Fish base
max_size <- length_to_weight(l = 200)  # From Fish base
com_size <- length_to_weight(l = common_length)
mat_size <- length_to_weight(l = maturation_length)
```

Parameters fitted by visual observation using consumption function

```
consumption_scalar <- 0.2
consumption_exp <- 0.635
```

Plot

Plot consumption_scalar and consumption_exp parameters to ensure compliance with user-defined fitting protocol.

```
  ggplot(data = data.frame(x = 0), mapping = aes(x = x)) +
    stat_function(fun = consumption, args = list(), size = 1, lty = "dashed",
                  aes(colour = "Max consumption")) +
    stat_function(fun = maintenance, args = list(), size = 1,
                  aes(colour = "Max maintenance")) +
    geom_vline(xintercept = mat_size, color = "red", linetype = "dashed") +
    geom_vline(xintercept = com_size, color = "blue", linetype = "dashed") +
    theme_bw() +
    labs(subtitle = paste(species), x = "Weight (g)", y = "Rate", color = "") +
    scale_color_viridis(discrete = TRUE) +
    scale_x_continuous(expand = c(0, 0), limits = c(0, max_size))
```

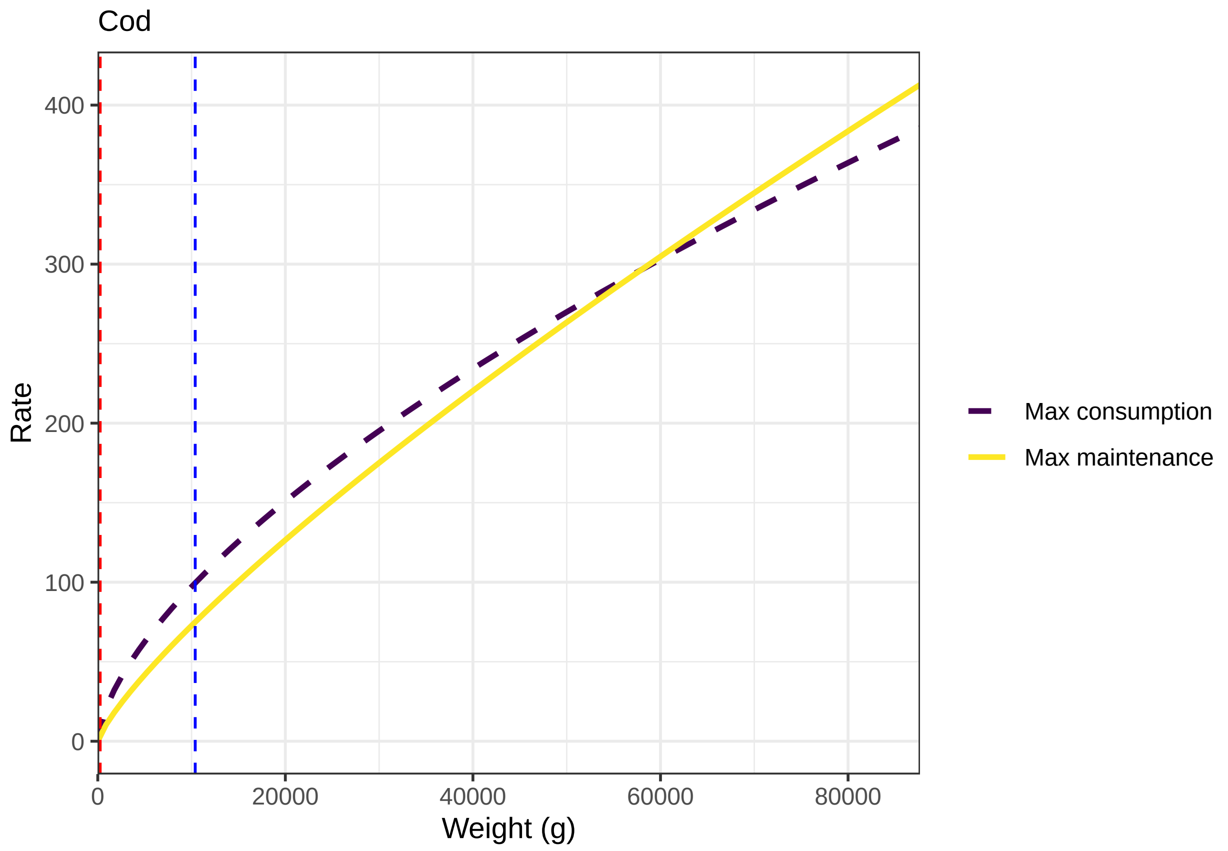

House keeping; clean environment for next species parameterization

```r
rm(list = setdiff(ls(), c("consumption", "maintenance", "length_to_weight", "weight_to_length")))
```

Thinlip mullet (*Chelon ramada*)

Parameters

```
species <- "Mullet"
weight_length_constant <- 4.48
weight_length_exp <- 0.341
QJ <- 0.8
allometric_scalar <- 0.03
allometric_exp <- 0.79
maturation_length <- 25.9
common_length <- 35  # From Fish base
max_size <- length_to_weight(l = 70)  # From Fish base
com_size <- length_to_weight(l = common_length)
mat_size <- length_to_weight(l = maturation_length)
```

Parameters fitted by visual observation using consumption function

```
consumption_scalar <- 0.3
consumption_exp <- 0.52
```

Plot

Plot consumption_scalar and consumption_exp parameters to ensure compliance with user-defined fitting protocol.

```
  ggplot(data = data.frame(x = 0), mapping = aes(x = x)) +
    stat_function(fun = consumption, args = list(), size = 1, lty = "dashed",
                  aes(colour = "Max consumption")) +
    stat_function(fun = maintenance, args = list(), size = 1,
                  aes(colour = "Max maintenance")) +
    geom_vline(xintercept = mat_size, color = "red", linetype = "dashed") +
    geom_vline(xintercept = com_size, color = "blue", linetype = "dashed") +
    theme_bw() +
    labs(subtitle = paste(species), x = "Weight (g)", y = "Rate", color = "") +
    scale_color_viridis(discrete = TRUE) +
    scale_x_continuous(expand = c(0, 0), limits = c(0, max_size))
```

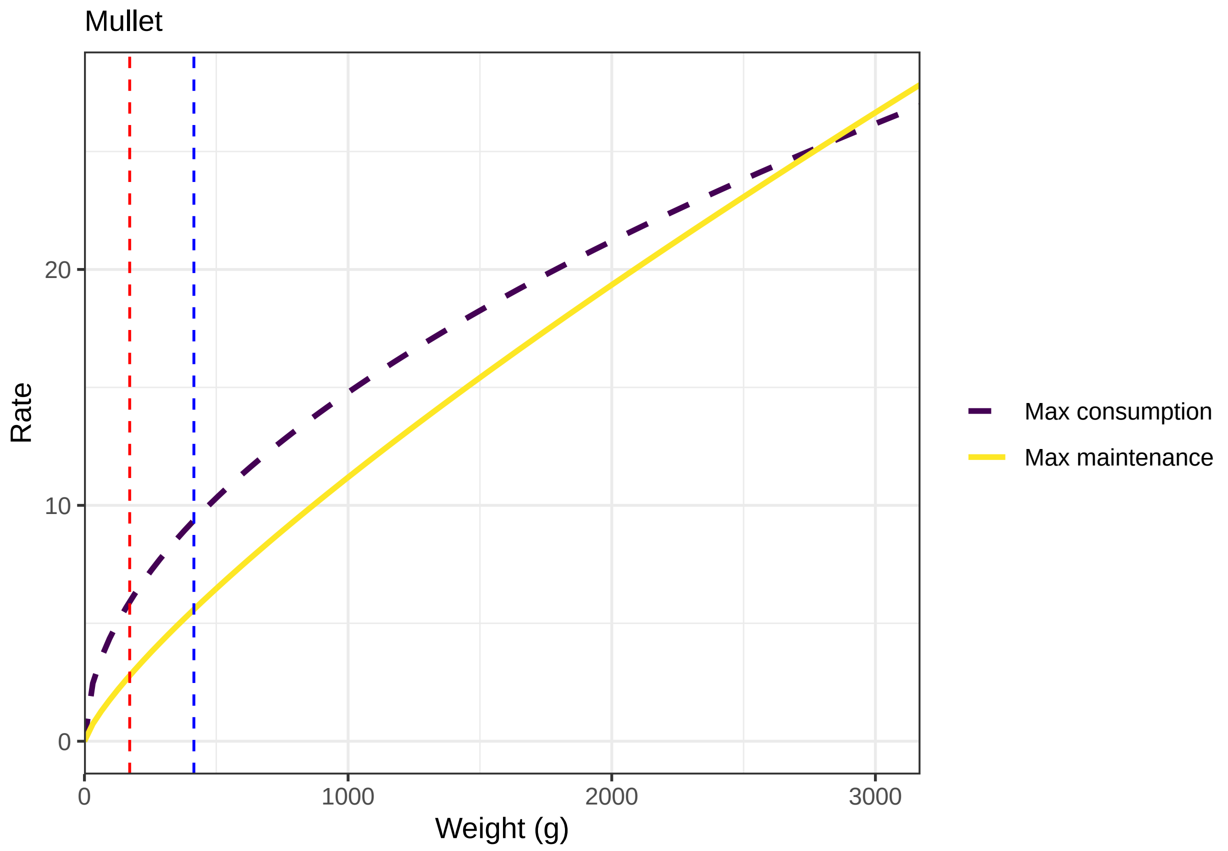

House keeping; clean environment for next species parameterization

```
rm(list = setdiff(ls(), c("consumption", "maintenance", "length_to_weight", "weight_to_length")))
```

2. Parameterization of the juvenile and maximum condition parameters

Juvenile Maximum Condition, q_j

The q_j parameter can be calculated from starvation experiments (e.g., McGurk (1984) DOI link).

The paper examines the maximum (maxw) and minimum (minw) weights of fish (focusing on average weights at the point of fish fallout).

We assume q_s = 0.2 and use this to calculate x, where:

$$x = minw/(1 + q_s)$$

From this, we compute q_j using the formula:

$$q_j = \frac{maxw}{x} - 1$$

This assumes that individuals do not change in structural mass x.

Adult Maximum Condition, q_a

Using Persson et al. (1998) as an example:

For the parameter q_j they assume a value of 0.742. Based on a gonadosomatic index (GSI) of 15%, they calculate q_a.

The GSI is defined as:

$$GSI = \frac{gonads}{total\ mass} \times 100\%$$

The assumption is that after spawning, individuals return to the maximum juvenile condition q_j, which is represented by:

$$w = (1 + q_j) \times x$$

Where w is the weight and x is the structural mass. The maximum adult weight is:

$$w = (1 + q_a) \times x$$

The investment in reproduction is therefore:

$$w_{postspawn} - w_{prespawn} = (1 + q_a) \times x - (1 + q_j) \times x = (q_a - q_j) \times x$$

This represents the weight of the gonads. Given the maximum GSI and maximum reproductive investment, we can calculate q_a.

The formula for GSI is:

$$GSI = \frac{(q_a - q_j) \times x}{(1 + q_a) \times x}$$

Thus, solving for q_a:

$$q_a = \frac{GSI + q_j}{1 - GSI}$$

Note that GSI is normally expressed as a percentage, but for the calculation, it is used as a fraction. In this paper, $q_j = 0.7$ and GSI = 0.15, which results in $q_a \approx 1$

3. Temperature dependent allometric scaler and exponent parameterization

Overview

- The allometric scaler and exponent for each temperature dependent function were best fit by the authors using the referenced data displayed in each plot. Data from various experimental approaches were utilized for the intake scaling based on the correlation between long-term temperature preference(s), optimum growth temperature(s) and lethal temperature(s), while respiration and activity experiments provided the necessary data for the maintenance scaling.
- In cases where experiment studies reported different values under varying experimental conditions (e..g. feeding regimes, pH, salinity), a strict statistical fitting procedure was not feasible. Instead, we used our best expert judgment to give greater weight to specific studies. This method is referenced as "Best fit by authors" in Table 3 of the main text.

Herring (*Clupea harengus*)

Maximum (lethal) temperature for growth

Function

```
herring_max_con <- function(length) {
  temp <- 23*length^(-0.0475)
  return(temp)
}
```

Plot

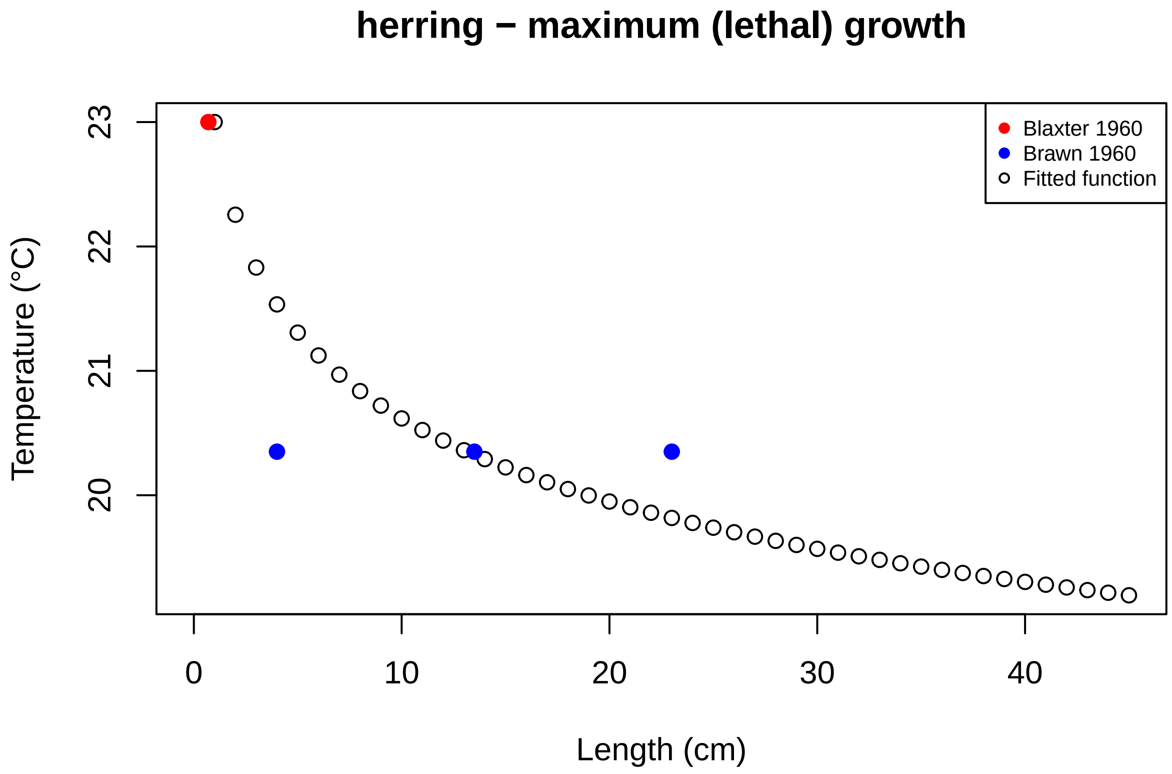

Optimal temperature for growth

Function

```
herring_opt_con <- function(length) {
  temp <- 17.0*length^(-0.031)
  return(temp)
}
```

Plot

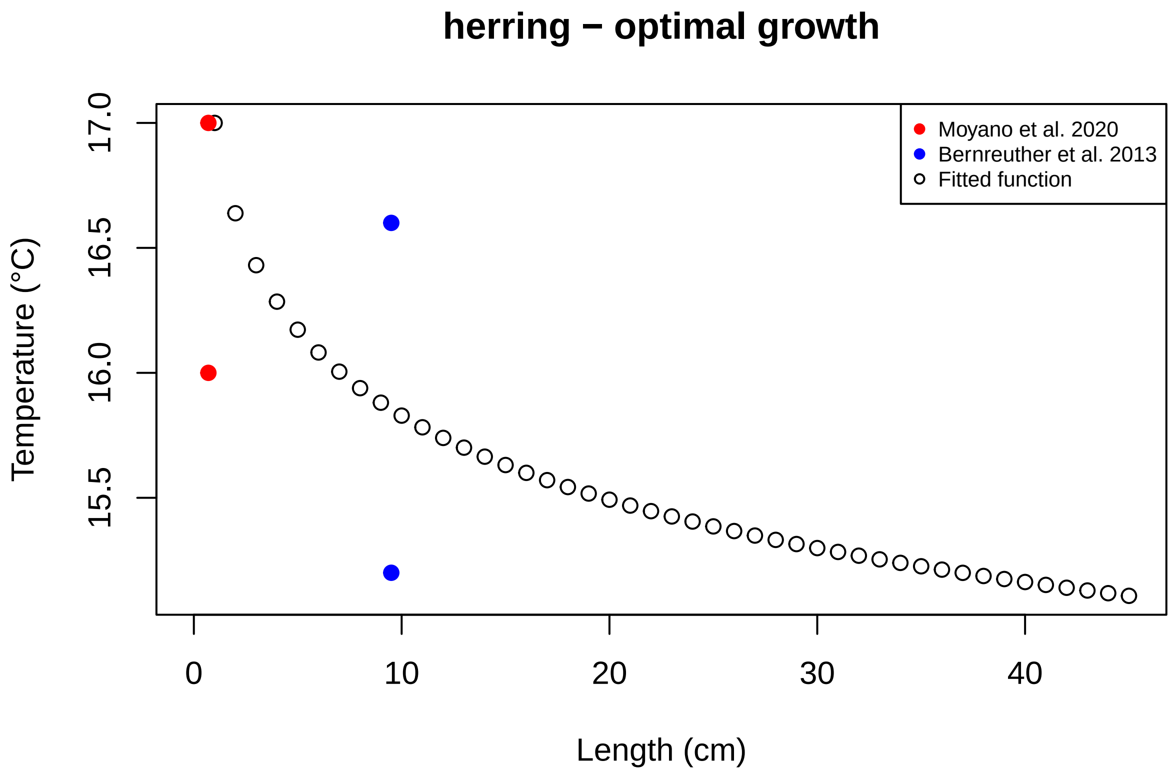

Maximum (lethal) temperature for metabolism

Function

```
herring_max_met <- function(length) {
  temp <- 24*length^(-0.05)
  return(temp)
}
```

Plot

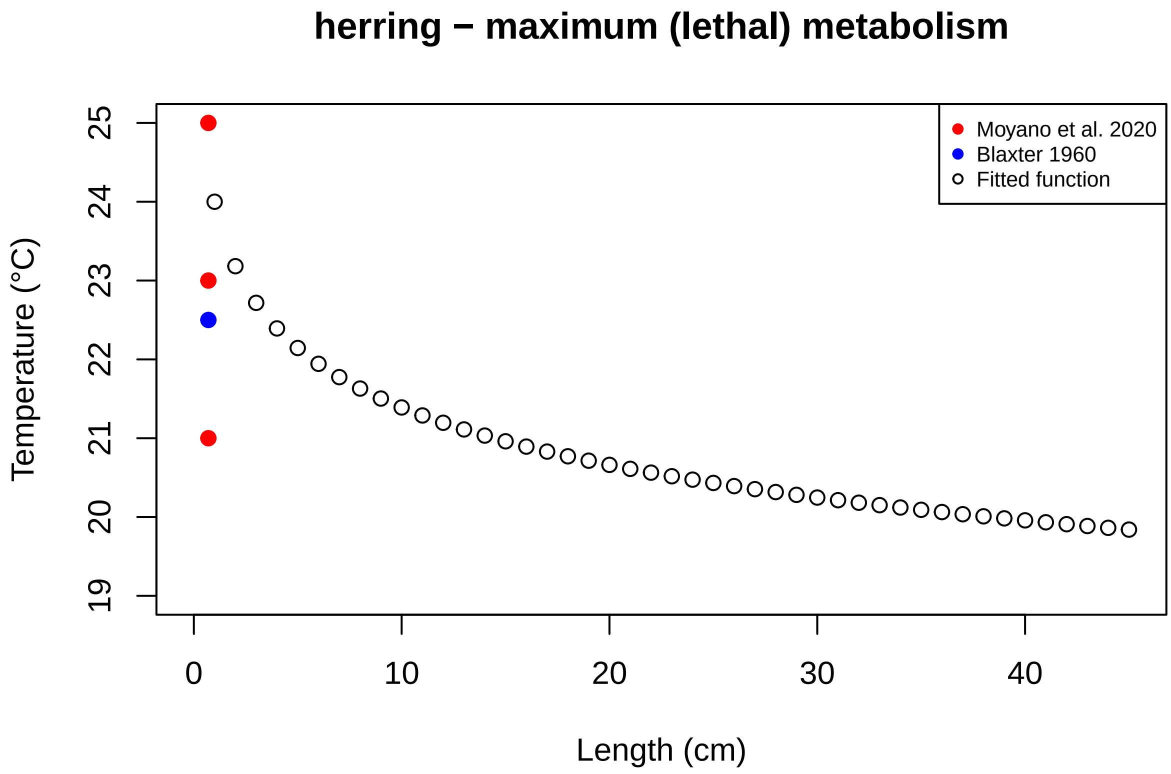

Optimal temperature for metabolism

Function

```
herring_opt_met <- function(length) {
  temp <- 16.5*length^(-0.028)
  return(temp)
}
```

Plot

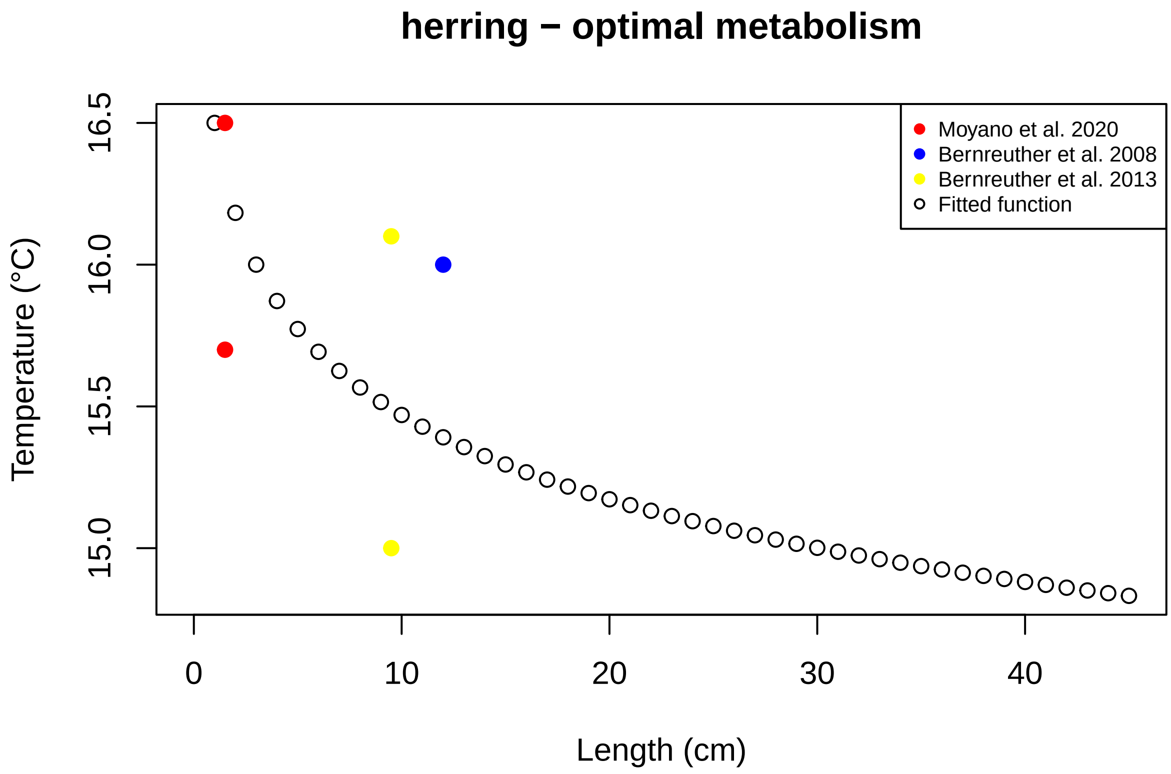

Sprat (*Sprattus sprattus*)

Maximum (lethal) temperature for growth

Function

```
sprat_max_con <- function(length) {
  temp <- 25*length^(-0.0475)
  return(temp)
}
```

Plot

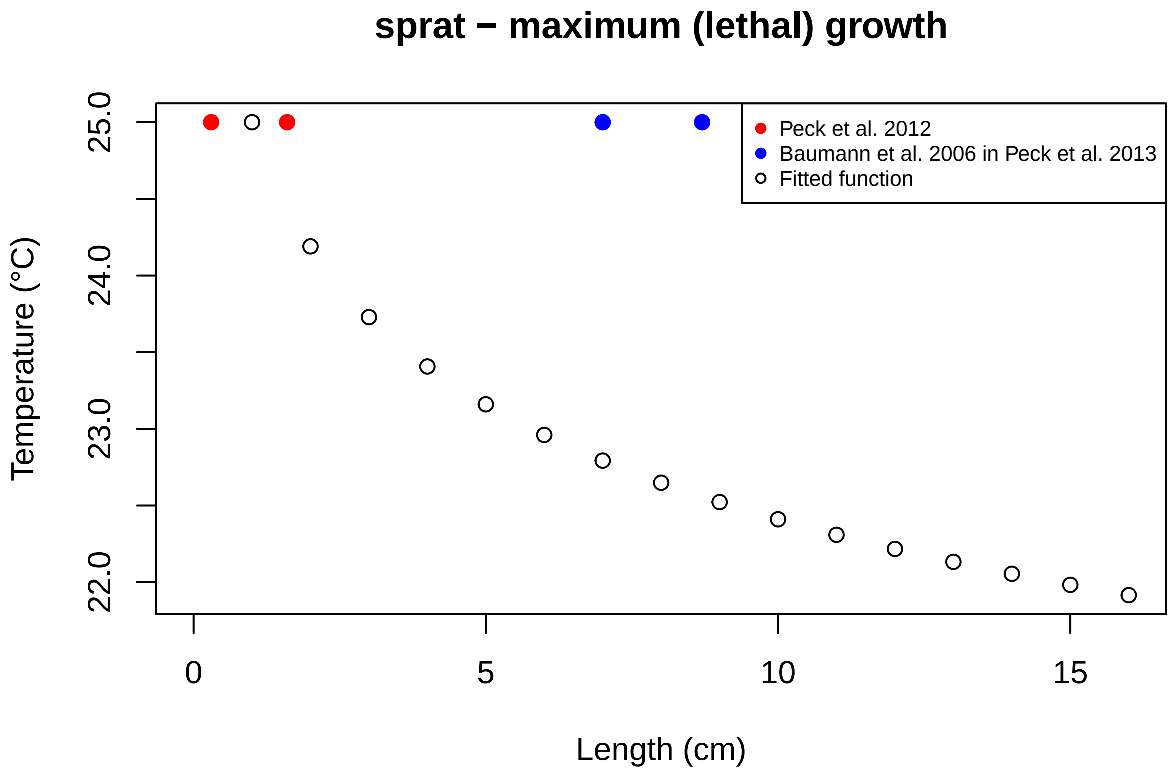

- Herring exponent used to fit curve.

Optimal temperature for growth

Function

```
sprat_opt_con <- function(length) {
  temp <- 21*length^(-0.055)
  return(temp)
}
```

Plot

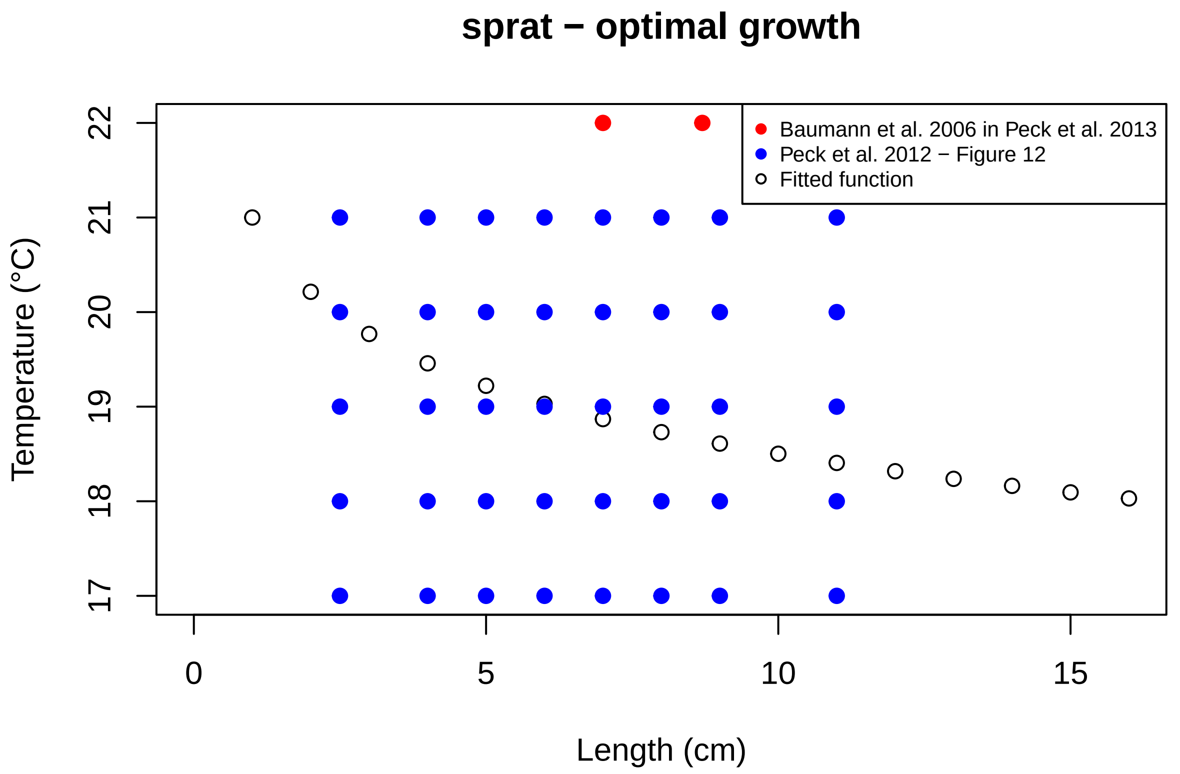

Maximum (lethal) temperature for metabolism

Function

```
sprat_max_met <- function(length) {
  temp <- 26.5*length^(-0.05)
  return(temp)
}
```

Plot

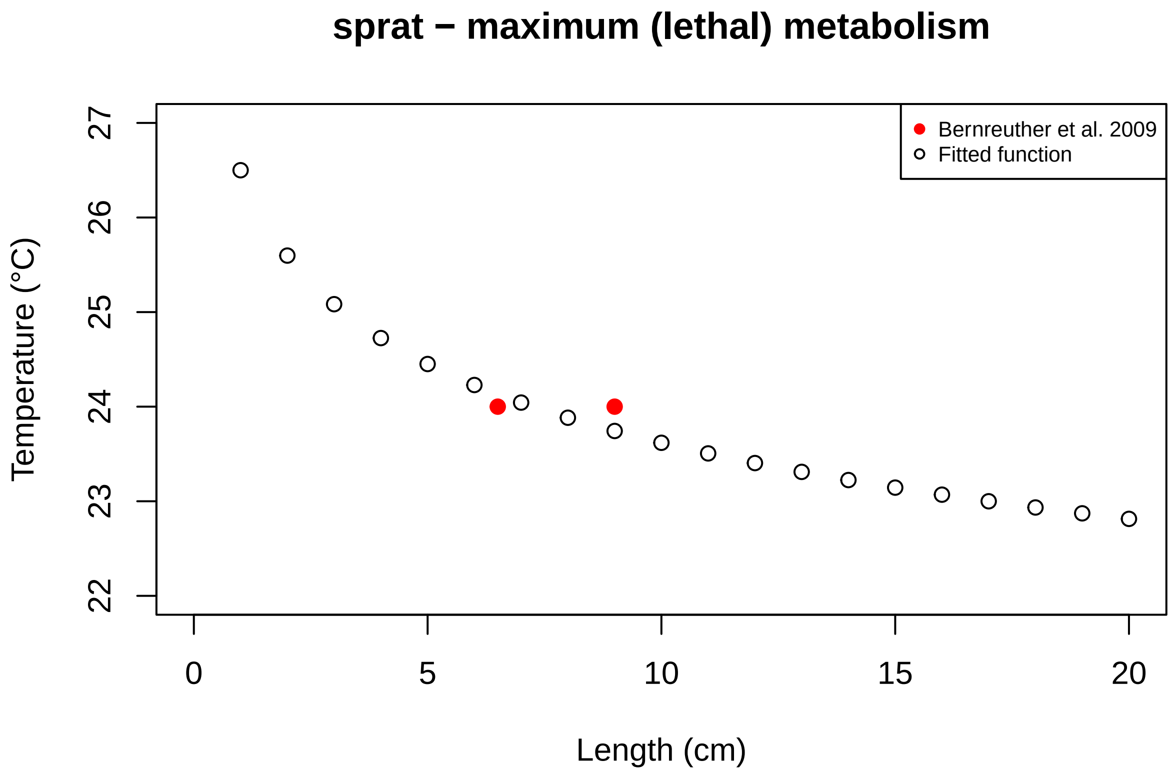

- Herring exponent used to fit curve.

Optimal temperature for metabolism

Function

```
sprat_opt_met <- function(length) {
  temp <- 20*length^(-0.028)
  return(temp)
}
```

Plot

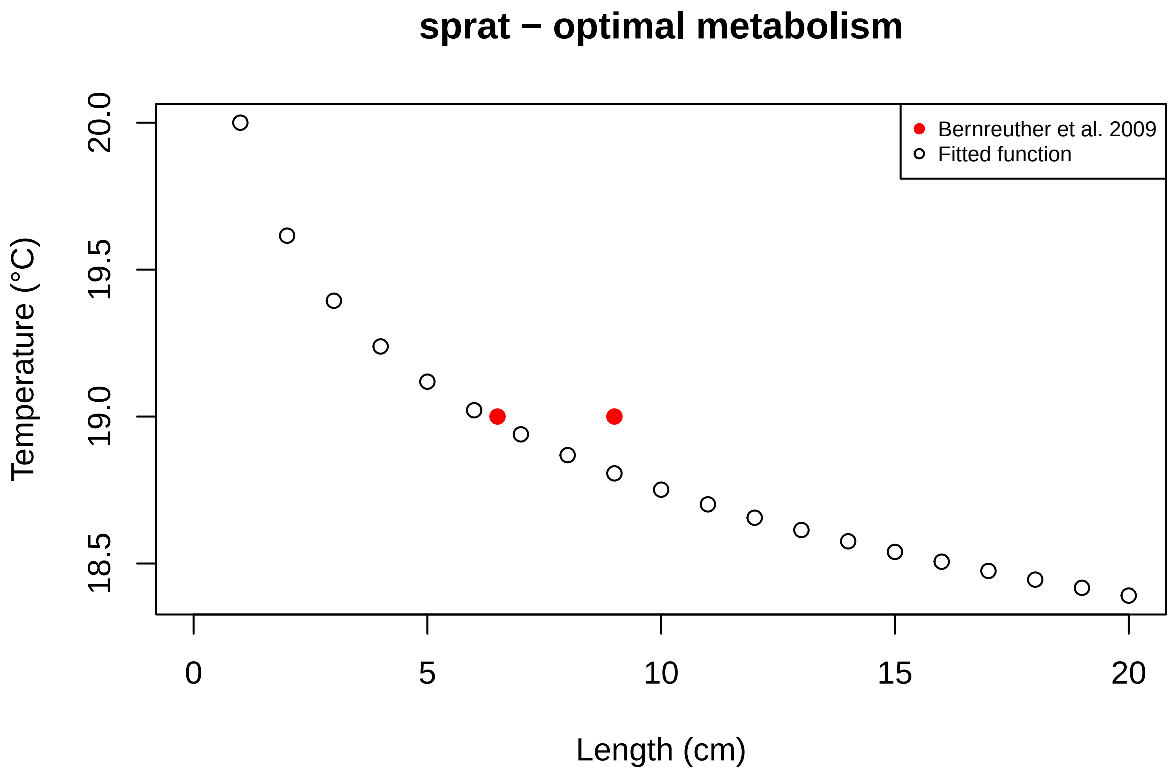

- Herring exponent used to fit curve.

Fivebeard rockling (*Ciliata mustela*)

Maximum (lethal) temperature for growth

Function

```
rockling_max_con <- function(length) {
  temp <- 27*length^(-0.0275)
  return(temp)
}
```

Plot

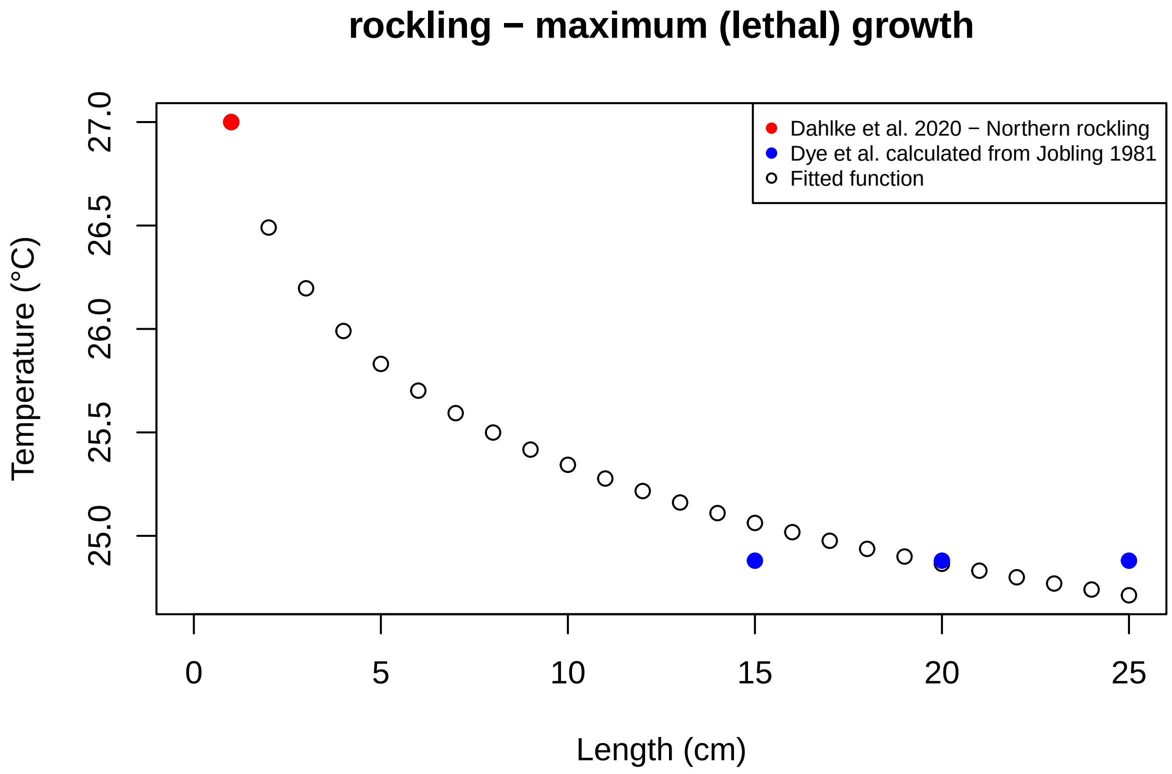

- (Dahlke et al. 2020) supplemental material provided thermal tolerance (Tmin, Tmax, Tmid, Trange) for the northern rockling (*Ciliata septentrionalis*).
- Mean temperature preference (12.88; Dye et al. 2024) from rockling experiments used in (Jobling 1981) table II equation to estimate maximum lethal temperature.

Optimal temperature for growth

Function

```
rockling_opt_con <- function(length) {
  temp <- 16.0*length^(-0.072)
  return(temp)
}
```

Plot

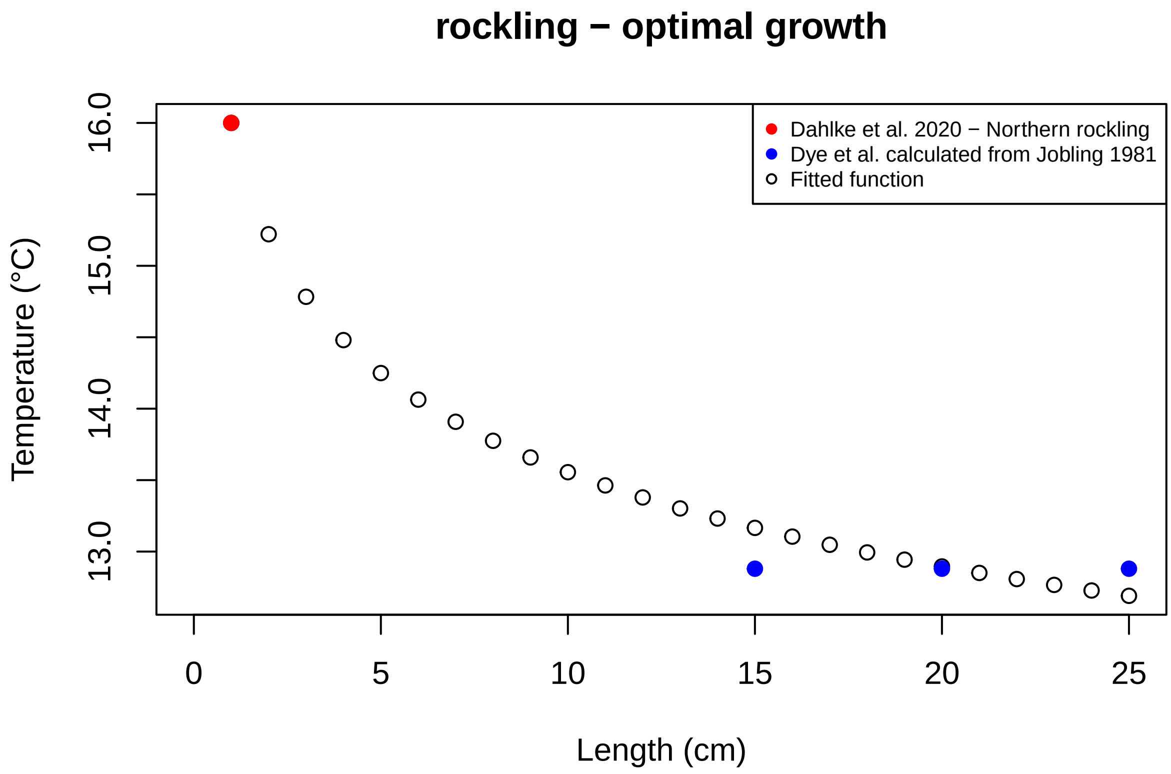

- (Dahlke et al. 2020) supplemental provided thermal tolerance (Tmin, Tmax, Tmid, Trange) for the northern rockling (*Ciliata septentrionalis*).
- Mean temperature preference (12.88; Dye et al. 2024) from rockling experiments used in (Jobling 1981) table II equation to estimate maximum lethal temperature.

Maximum (lethal) temperature for metabolism

Function

```
rockling_max_met <- function(length) {
  temp <- 29*length^(-0.029)
  return(temp)
}
```

Plot

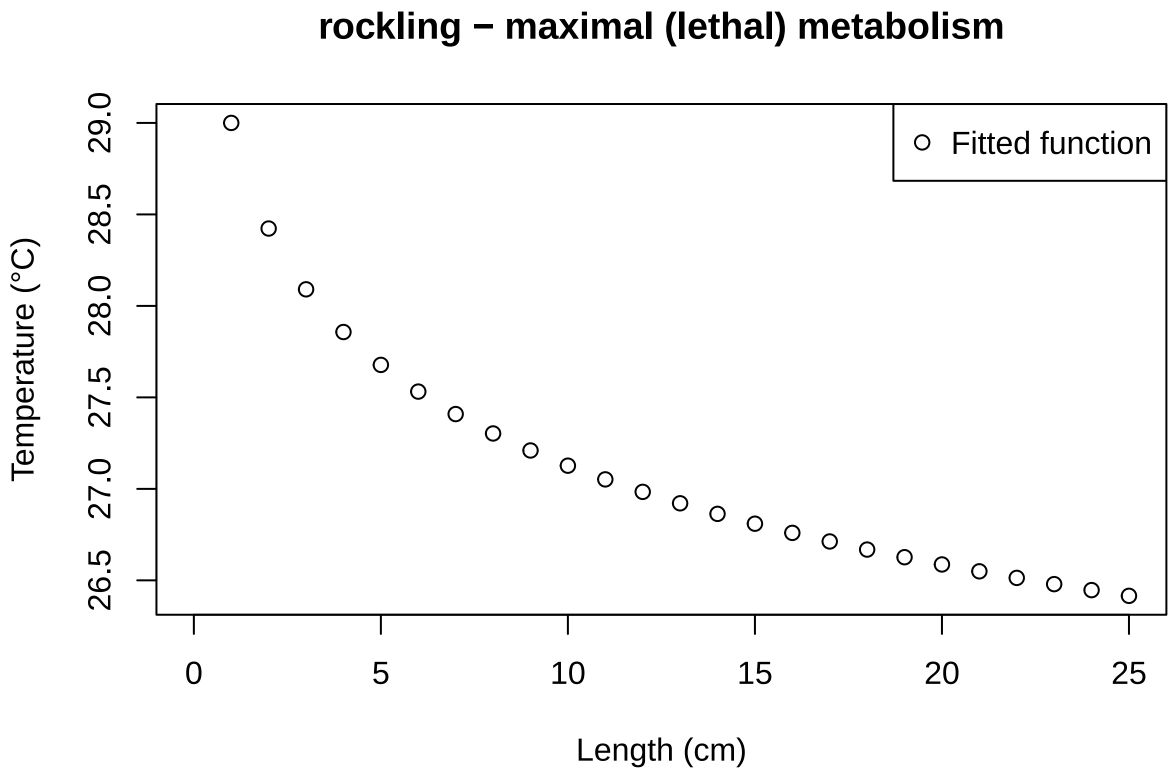

Optimal temperature for metabolism

Function

```
rockling_opt_met <- function(length) {
  temp <- 17.5*length^(-0.025)
  return(temp)
}
```

Plot

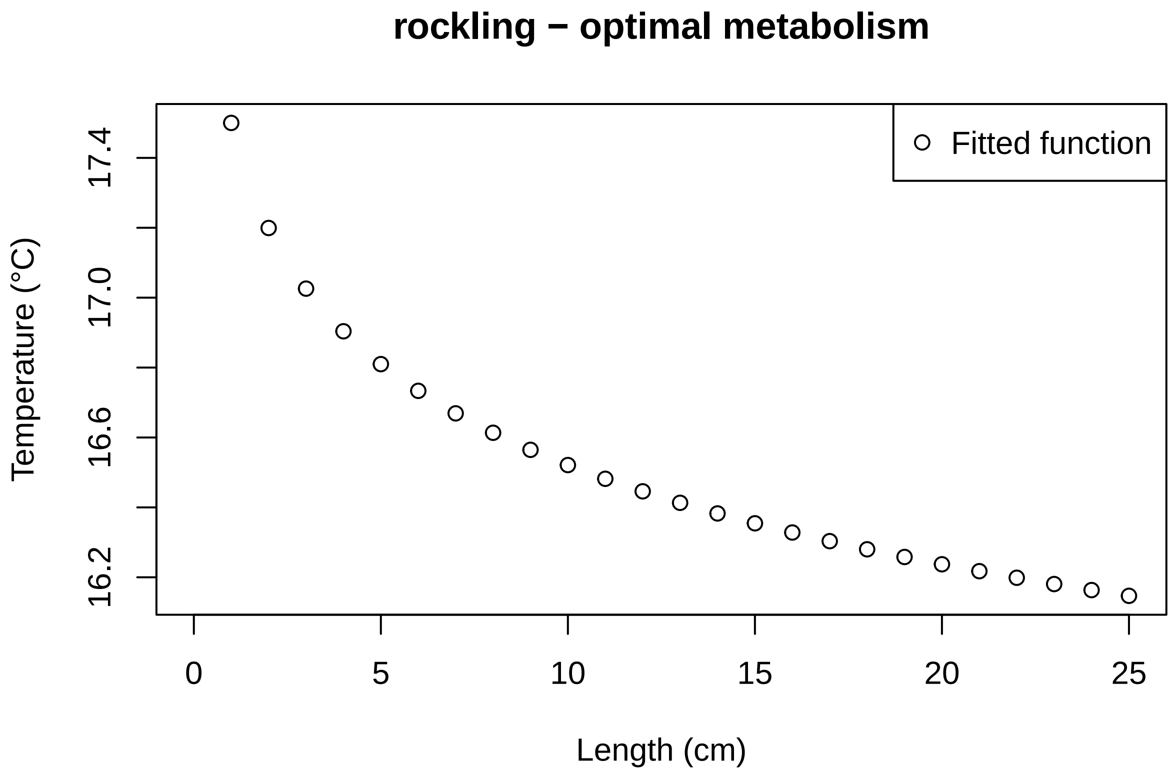

Cod (*Gadus morhua*)

Maximum (lethal) temperature for growth

Function

```
cod_max_con <- function(weight) {
  temp <- 22*weight^(-0.0435)
  return(temp)
}
```

Plot

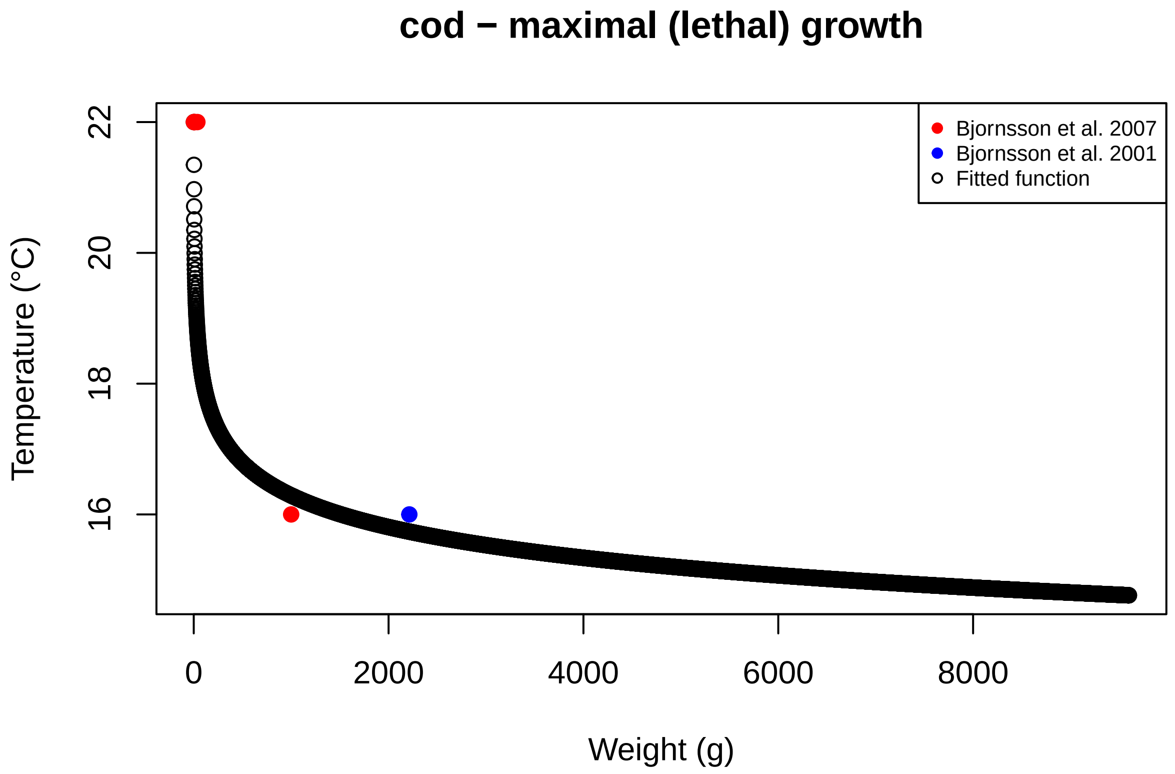

Optimal temperature for growth

Function - (Björn Björnsson, Steinarsson, and Árnason 2007)

```
cod_Bjorn <- function(W) {
  topt = 15.57-0.8426*log(W)
  return(topt)
}
```

Function

```
cod_opt_con <- function(weight) {
  temp <- 16.78*weight^(-0.078)
  return(temp)
}
```

- Equation from (Björn Björnsson, Steinarsson, and Árnason 2007) was rearranged and put into power regression form to obtain scalar and exponent.

Plot

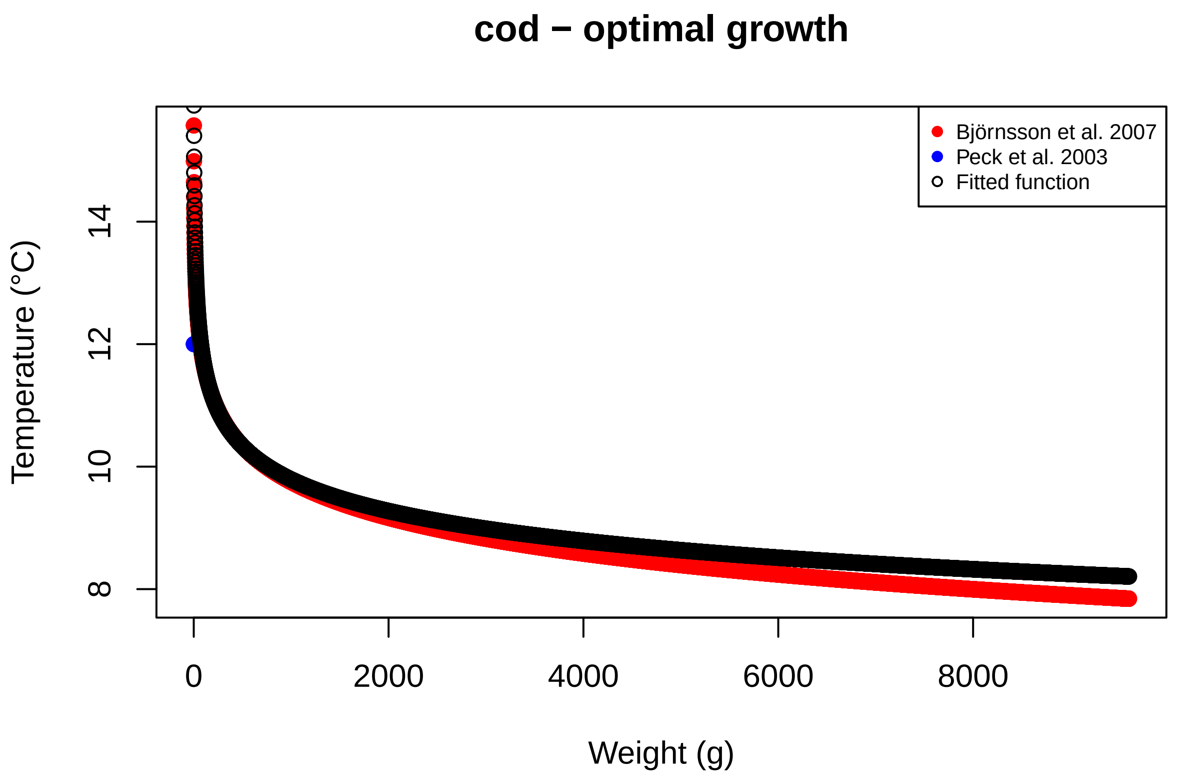

Maximum (lethal) temperature for metabolism

Function

```
cod_max_met <- function(weight) {
  temp <- 24*weight^(-0.029)
  return(temp)
}
```

Plot

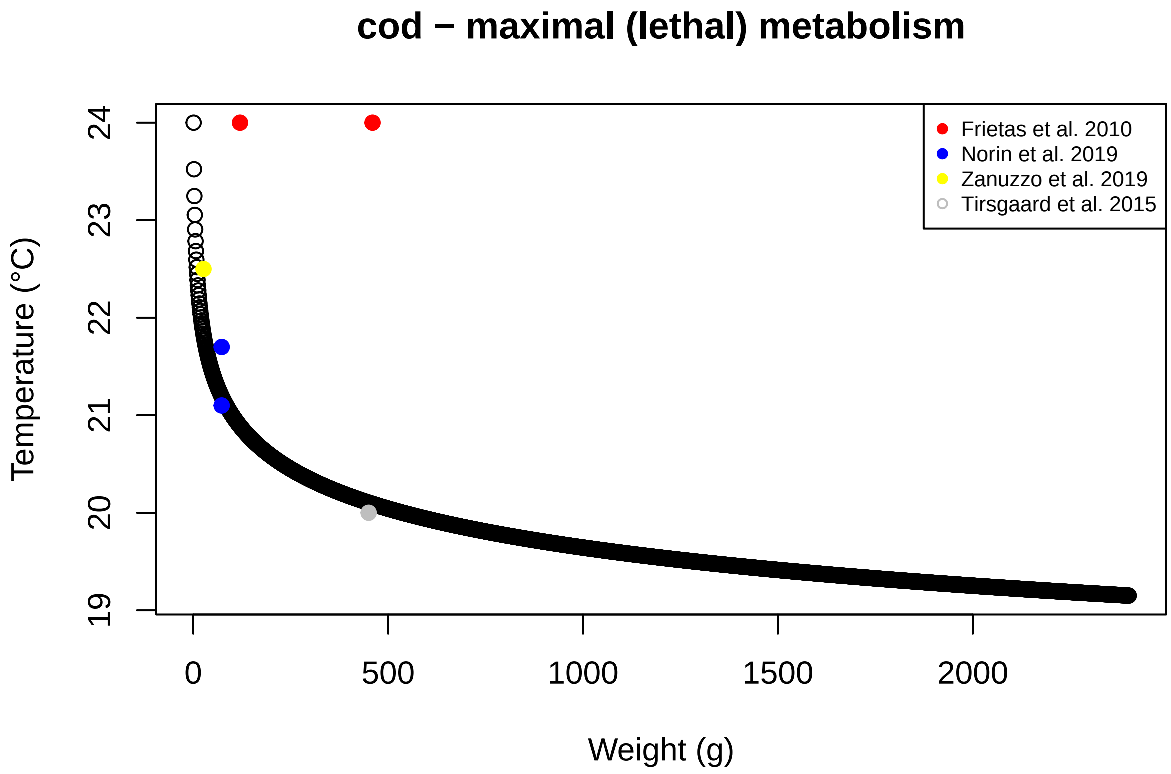

Optimal temperature for metabolism

Function

```r
cod_opt_met <- function(weight) {
  temp <- 15.5*weight^(-0.02)
  return(temp)
}
```

Plot

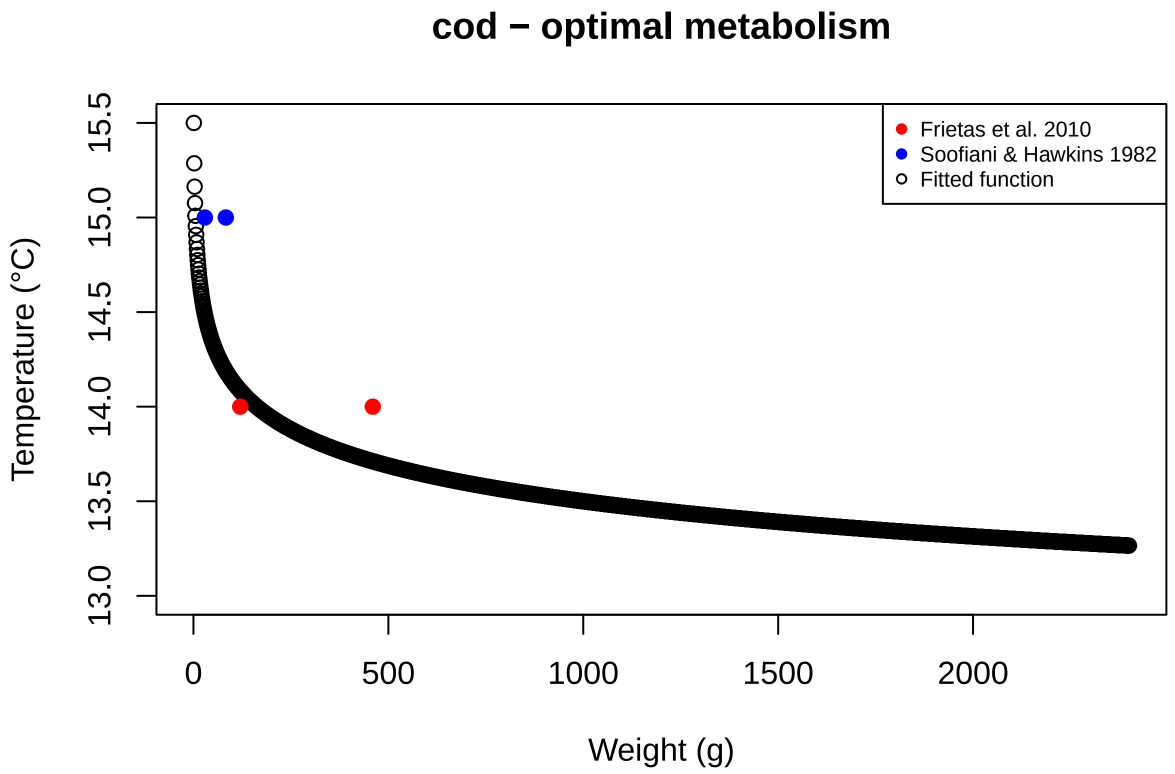

Thinlip mullet (*Chelon ramada*)

Maximum (lethal) temperature for growth

Function

```
mullet_max_con <- function(weight) {
  temp <- 37*weight^(-0.031)
  return(temp)
}
```

Plot

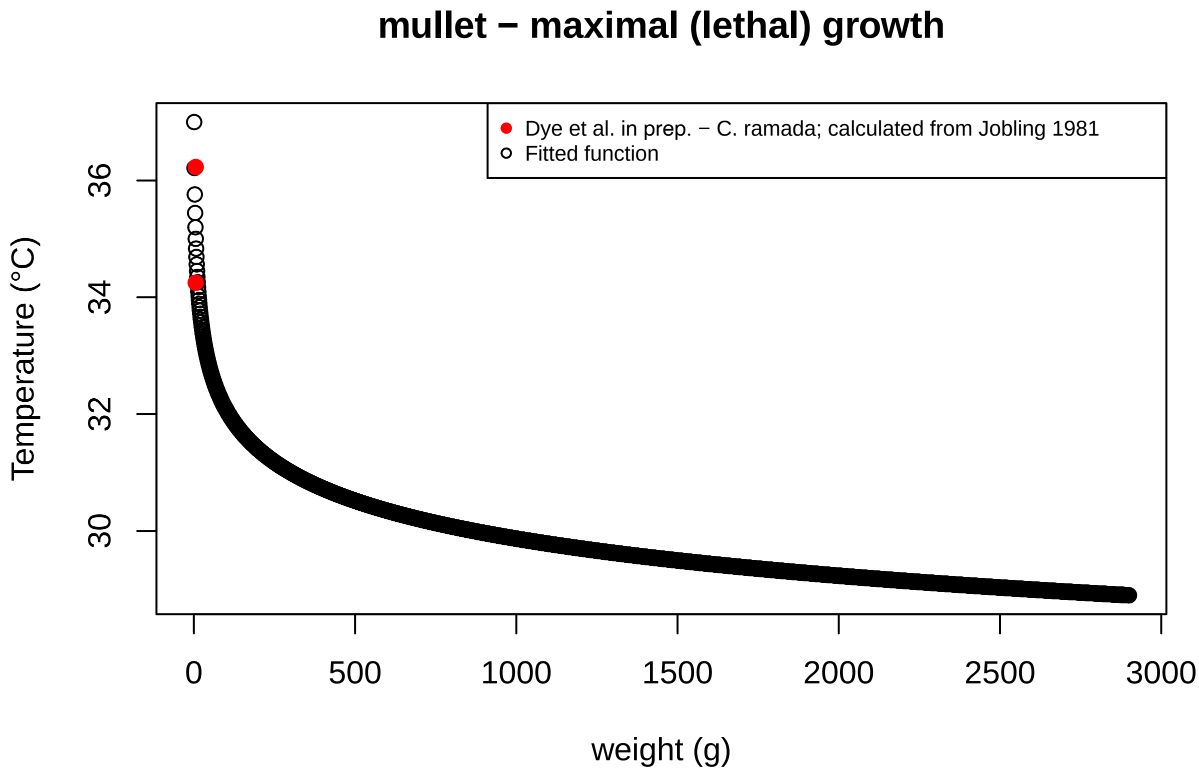

- Mullet experiments temperature preference (27-30*C; mean = 5.8 g, 8.1 cm; Kuijten 2022; Dye et al. in prep) used in (Jobling 1981) table II equation to estimate lethal (max) temperature.

Optimal temperature for growth

Function

```
mullet_opt_con <- function(weight) {
  temp <- 30*weight^(-0.041)
  return(temp)
}
```

Plot

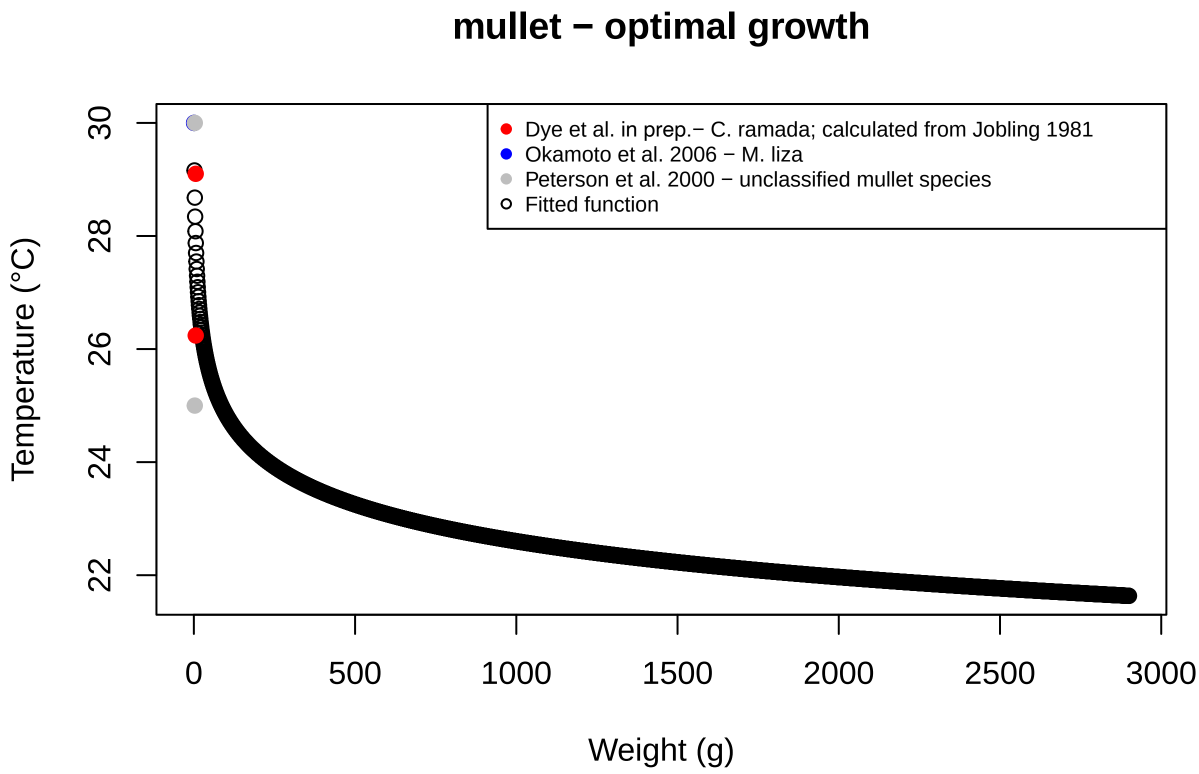

- Mullet experiments temperature preference (27-30*C; mean = 5.8 g, 8.1 cm; Kuijten 2022; Dye et al. in prep) used in (Jobling 1981) table II equation to estimate optimal temperature.

Maximum temperature for metabolism

Function

```
mullet_max_met <- function(weight) {
  temp <- 42*weight^(-0.0295)
  return(temp)
}
```

Plot

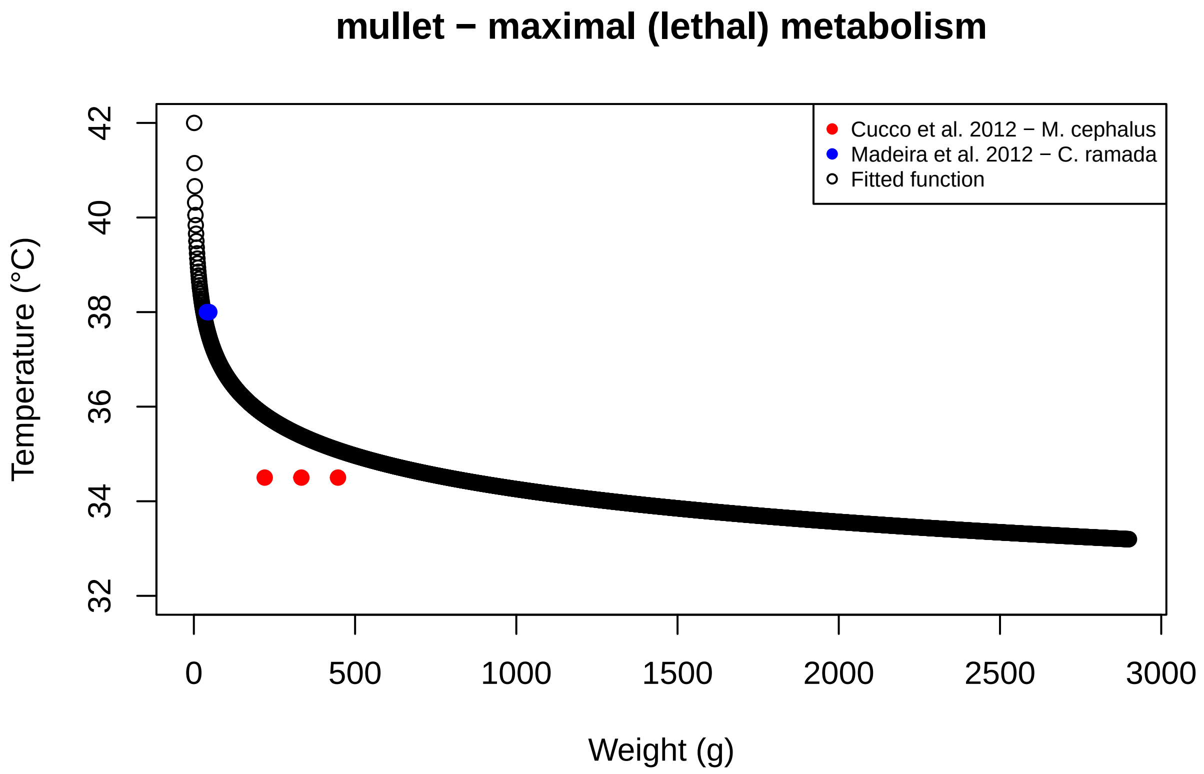

Optimal temperature for metabolism

Function

```
mullet_opt_met <- function(weight) {
  temp <- 34*weight^(-0.04)
  return(temp)
}
```

Plot

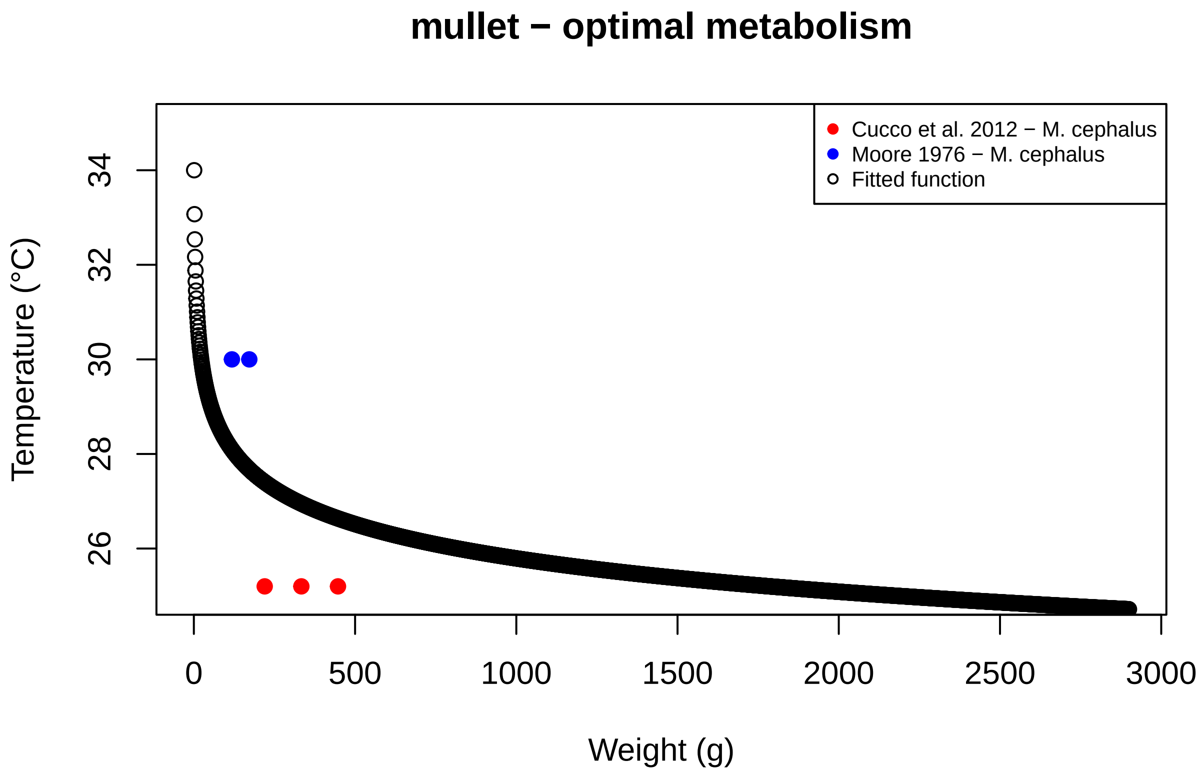

4. Temperature dependent scaling

To scale the temperature dependence of physiological rates (maintenance and intake rates), the default, size-dependent value of each physiological rate is multiplied by a temperature dependent factor (r_a, r_m - Table 3; Figure 1). We formulate the temperature adjustment factors for each physiological process accounting for size specific temperature tolerance (lethal) ranges and optimum temperatures (Karås & Thoresson, 1992; Ohlberger et al., 2011). Figures 1 and 2 illustrate the calculated temperature dependent factors for a non-specific fish species (similar to European perch, *Perca fluviatilis*).

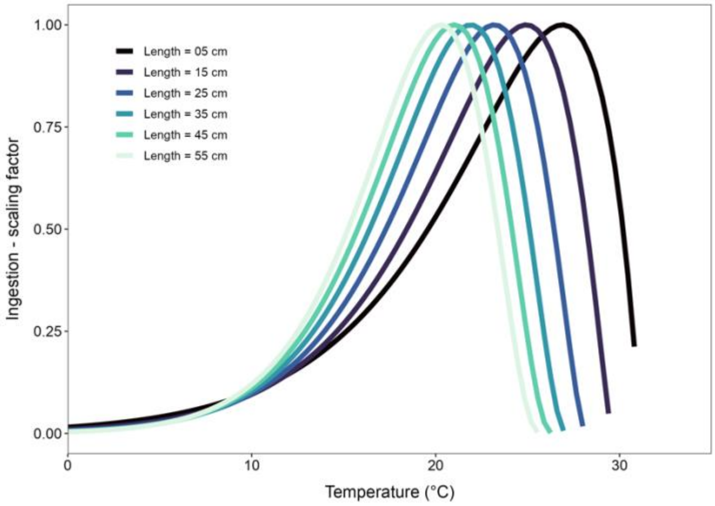

Figure 1. Temperature dependent ingestion scaler.

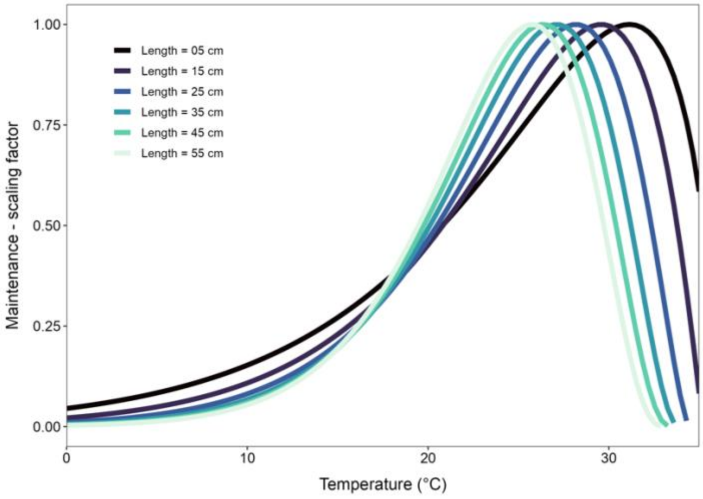

Figure 2. Temperature dependent maintenance scaler.

5. Lifetime fecundity during contrasting environmental conditions

Lifetime fecundity (number of eggs produced per individual) predictions for herring and rockling. Reproduction occurs on the first day of the model year and at that time all accumulated gonadal mass was converted into the number of eggs, F(x, y), described by the fecundity equation (Table 5) which accounts for the reproduction efficiency (k_r) (i.e. gonad-offspring conversion) and the species-specific egg mass (w_b). The figure shows discrete spawning events (points), with labels indicating the predicted total length of individuals. A horizontal line between points indicates that the fish was alive but did not spawn.

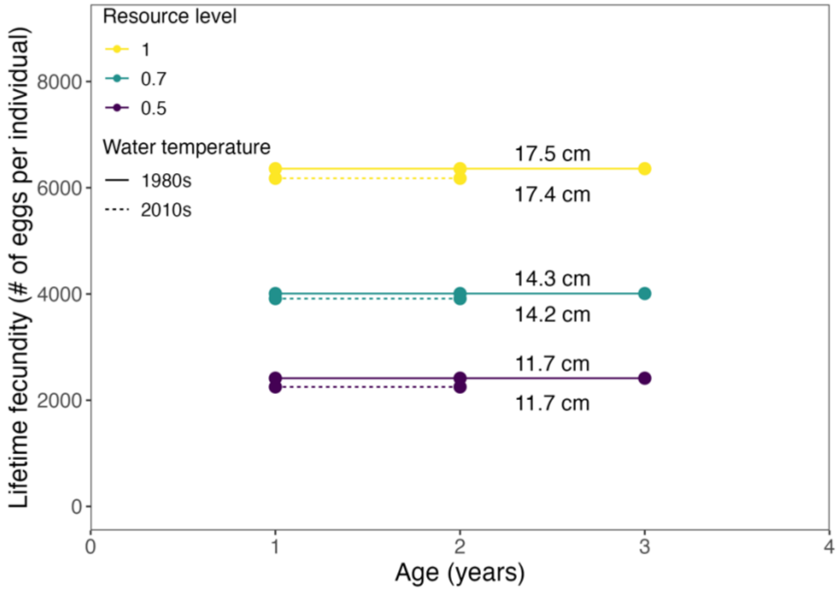

Figure 1. The influence of contrasting environmental conditions on rockling model predicted lifetime fecundity (# eggs per individual).

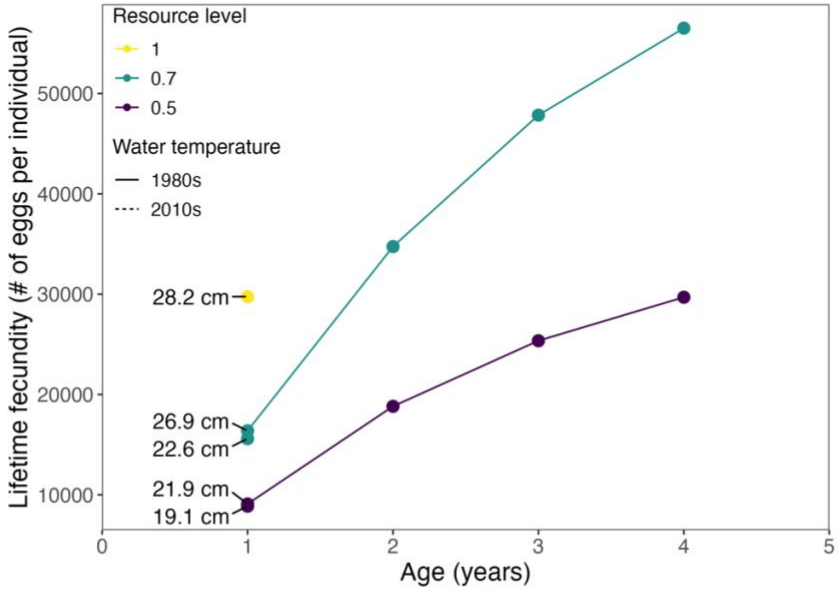

Figure 2. The influence of contrasting environmental conditions on herring model predicted lifetime fecundity (# eggs per individual).

References

Baumann, H, T Gröhsler, G Kornilovs, A Makarchouk, V Feldmann, and A Temming. 2006. "Temperature-Induced Regional and Temporal Growth Differences in Baltic Young-of-the-Year Sprat Sprattus Sprattus." *Marine Ecology Progress Series* 317 (July): 225–36. https://doi.org/10.3354/meps317225.

Bernreuther, M., J.-P. Herrmann, M. A. Peck, and A. Temming. 2012. "Growth Energetics of Juvenile Herring, *Clupea Harengus* L.: Food Conversion Efficiency and Temperature Dependency of Metabolic Rate." *Journal of Applied Ichthyology* 29 (2): 331–40. https://doi.org/10.1111/jai.12045.

Bernreuther, M., J.-P. Herrmann, and A. Temming. 2008. "Laboratory Experiments on the Gastric Evacuation of Juvenile Herring (Clupea Harengus L.)." *Journal of Experimental Marine Biology and Ecology* 363 (1-2): 1–11. https://doi.org/10.1016/j.jembe.2008.05.012.

Bernreuther, M., A. Temming, and J.-P. Herrmann. 2009. "Effect of Temperature on the Gastric Evacuation in Sprat*Sprattus Sprattus*." *Journal of Fish Biology* 75 (7): 1525–41. https://doi.org/10.1111/j.1095-8649.2009.02353.x.

Björnsson, B. 2001. "Optimal Temperature for Growth and Feed Conversion of Immature Cod (Gadus Morhua L.)." *ICES Journal of Marine Science* 58 (1): 29–38. https://doi.org/10.1006/jmsc.2000.0986.

Björnsson, Björn, Agnar Steinarsson, and Tómas Árnason. 2007. "Growth Model for Atlantic Cod (Gadus Morhua): Effects of Temperature and Body Weight on Growth Rate." *Aquaculture* 271 (1-4): 216–26. https://doi.org/10.1016/j.aquaculture.2007.06.026.

Blaxter, J. H. S. 1960. "The Effect of Extremes of Temperature on Herring Larvae." *Journal of the Marine Biological Association of the United Kingdom* 39 (3): 605–8. https://doi.org/10.1017/s0025315400013576.

Brawn, Vivien M. 1960. "Temperature Tolerance of Unacclimated Herring (*Clupea Harengus* L.)." *Journal of the Fisheries Research Board of Canada* 17 (5): 721–23. https://doi.org/10.1139/f60-057.

Cucco, Andrea, Matteo Sinerchia, Christel Lefrançois, Paolo Magni, Michol Ghezzo, Georg Umgiesser, Angelo Perilli, and Paolo Domenici. 2012. "A Metabolic Scope Based Model of Fish Response to Environmental Changes." *Ecological Modelling* 237-238 (July): 132–41. https://doi.org/10.1016/j.ecolmodel.2012.04.019.

Dahlke, Flemming T., Sylke Wohlrab, Martin Butzin, and Hans-Otto Pörtner. 2020. "Thermal Bottlenecks in the Life Cycle Define Climate Vulnerability of Fish." *Science* 369 (6499): 65–70. https://doi.org/10.1126/science.aaz3658.

Dye, B., Kuijten S., Tulp. I., Blom E., Poos J. J., and E. Schram. in prep.. "Influence of Salinity on the Thermal Preference of Juvenile Thinlip Mullet, Chelon Ramada." in preparation for *Journal of Experimental Marine Biology and Ecology.*

Dye, B., Tulp. I., van Leeuwen A., Blom E., and E. Schram. 2024. "A Rockling's Choice: The Trade-Off Between Thermal Preference and Physical Structure in the Five Bearded Rockling, Ciliata Mustela." *Journal of Experimental Marine Biology and Ecology.*

Freitas, Vânia, Joana F. M. F. Cardoso, Konstadia Lika, Myron A. Peck, Joana Campos, Sebastiaan A. L. M. Kooijman, and Henk W. van der Veer. 2010. "Temperature Tolerance and Energetics: A Dynamic Energy Budget-Based Comparison of North Atlantic Marine Species." *Philosophical Transactions of the Royal Society B: Biological Sciences* 365 (1557): 3553–65. https://doi.org/10.1098/rstb.2010.0049.

Jobling, M. 1981. "Temperature Tolerance and the Final Preferendum-Rapid Methods for the Assessment of Optimum Growth Temperatures." *Journal of Fish Biology* 19 (4): 439–55. https://doi.org/10.1111/j.1095-8649.1981.tb05847.x.

Karås, P., and G. Thoresson. 1992. "An Application of a Bioenergetics Model to Eurasian Perch (Perca Fluviatilis L.)." *Journal of Fish Biology*, 217–30.

Kuitjen, S. 2022. "Identifying Thermal Preference of Golden Grey Mullet." *Masters Thesis*, 1–31.

Madeira, Diana, Luís Narciso, Henrique N. Cabral, and Catarina Vinagre. 2012. "Thermal Tolerance and Potential Impacts of Climate Change on Coastal and Estuarine Organisms." *Journal of Sea Research* 70 (May): 32–41. https://doi.org/10.1016/j.seares.2012.03.002.

Moore, R. H. 1976. "Seasonal Patterns in the Respiratory Metabolism of Mullets Mugil Cephalus and Mugil Curema." *Contributions in Marine Science* 20: 133–46.

Moyano, Marta, Björn Illing, Patrick Polte, Paul Kotterba, Yury Zablotski, Tomas Gröhsler, Patricia Hüdepohl, Steven J. Cooke, and Myron A. Peck. 2020. "Linking Individual Physiological Indicators to the Productivity of Fish Populations: A Case Study of Atlantic Herring." *Ecological Indicators* 113 (June): 106146. https://doi.org/10.1016/j.ecolind.2020.106146.

Norin, Tommy, Paula Canada, Jason A. Bailey, and A. Kurt Gamperl. 2019. "Thermal Biology and Swimming Performance of Atlantic Cod (*Gadus Morhua*) and Haddock (*Melanogrammus Aeglefinus*)." *PeerJ* 7 (October): e7784. https://doi.org/10.7717/peerj.7784.

Ohlberger, Jan, Eric Edeline, Leif Asbjørn Vøllestad, Nils C. Stenseth, and David Claessen. 2011. "Temperature-Driven Regime Shifts in the Dynamics of Size-Structured Populations." *The American Naturalist* 177 (2): 211–23. https://doi.org/10.1086/657925.

Okamoto, Marcelo Hideo, Luís André Sampaio, and Armindo De Pinho Maçada. 2006. "Efeito Da Temperatura Sobre o Crescimento e a Sobrevivência de Juvenis Da Tainha Mugil Platanus Günther, 1880." *Atlântica, Rio Grande* 28: 61–66. https://repositorio.furg.br/bitstream/handle/1/693/EFEITO%20DA%20TEMPERATURA%20SOBRE%20O%20CRESCIMENTO%20E%20A%20SOBREVIV%CANCIA.pdf?sequence=1.

Peck, M. A., L. J. Buckley, E. M. Caldarone, and D. A. Bengtson. 2003. "Effects of Food Consumption and Temperature on Growth Rate and Biochemical-Based Indicators of Growth in Early Juvenile Atlantic Cod Gadus Morhua and Haddock Melanogrammus Aeglefinus." *Marine Ecology Progress Series* 251: 233–43.

Peck, Myron A., Hannes Baumann, Matthias Bernreuther, Catriona Clemmesen, Jens-Peter Herrmann, Holger Haslob, Bastian Huwer, et al. 2012. "The Ecophysiology of Sprattus Sprattus in the Baltic and North Seas." *Progress in Oceanography* 103 (September): 42–57. https://doi.org/10.1016/j.pocean.2012.04.013.

Peck, Myron A., Patricia Reglero, Motomitsu Takahashi, and Ignacio A. Catalán. 2013. "Life Cycle Ecophysiology of Small Pelagic Fish and Climate-Driven Changes in Populations." *Progress in Oceanography* 116 (September): 220–45. https://doi.org/10.1016/j.pocean.2013.05.012.

Persson, Lennart, Kjell Leonardsson, André M. de Roos, Mats Gyllenberg, and Bent Christensen. 1998. "Ontogenetic Scaling of Foraging Rates and the Dynamics of a Size-Structured Consumer-Resource Model." *Theoretical Population Biology* 54 (3): 270–93. https://doi.org/10.1006/tpbi.1998.1380.

Peterson, M. S., Rakocinski C. F., Comyns B. H., and G. L. Fulling. 2000. "Laboratory Growth Responses of Juvenile Mugil Sp. To Temperature and Salinity: Delineating Optimal Field Growth Conditions." *Proceedings of the Gulf and Caribbean Fisheries Institute, 51*, 341–52. http://hdl.handle.net/1834/29305.

Soofiani, N. M., and A. D. Hawkins. 1982. "Energetic Costs at Different Levels of Feeding in Juvenile Cod, Gadus Morhua L." *Journal of Fish Biology* 21 (5): 577–92. https://doi.org/10.1111/j.1095-8649.1982.tb02861.x.

Tirsgaard, Bjørn, Jane W. Behrens, and John F. Steffensen. 2015. "The Effect of Temperature and Body Size on Metabolic Scope of Activity in Juvenile Atlantic Cod Gadus Morhua L." *Comparative Biochemistry and Physiology Part A: Molecular & Integrative Physiology* 179 (January): 89–94. https://doi.org/10.1016/j.cbpa.2014.09.033.

Zanuzzo, Fábio S., Jason A. Bailey, Amber F. Garber, and Anthony K. Gamperl. 2019. "The Acute and Incremental Thermal Tolerance of Atlantic Cod (Gadus Morhua) Families Under Normoxia and Mild Hypoxia." *Comparative Biochemistry and Physiology Part A: Molecular & Integrative Physiology* 233 (July): 30–38. https://doi.org/10.1016/j.cbpa.2019.03.020.
